# A 3D Epithelial–Mesenchymal Co-Culture Model of the Airway Wall Using Native Lung Extracellular Matrix

**DOI:** 10.3390/bioengineering11090946

**Published:** 2024-09-21

**Authors:** Roderick H. J. de Hilster, Marjan A. Reinders-Luinge, Annemarie Schuil, Theo Borghuis, Martin C. Harmsen, Janette K. Burgess, Machteld N. Hylkema

**Affiliations:** 1 Department of Pathology and Medical Biology, University of Groningen, University Medical Center Groningen, 9713 GZ Groningen, The Netherlands; r.h.j.de.hilster@umcg.nl (R.H.J.d.H.);; 2 Groningen Research Institute for Asthma and COPD (GRIAC), University of Groningen, University Medical Center Groningen, 9713 GZ Groningen, The Netherlands; 3 KOLFF Institute—REGENERATE, University of Groningen, University Medical Center Groningen, FB41, 9713 AV Groningen, The Netherlands

**Keywords:** extracellular matrix, COPD, TWOMBLI

## Abstract

Chronic obstructive pulmonary disease (COPD) is a chronic lung disease characterized by ongoing inflammation, impaired tissue repair, and aberrant interplay between airway epithelium and fibroblasts, resulting in an altered extracellular matrix (ECM) composition. The ECM is the three-dimensional (3D) scaffold that provides mechanical support and biochemical signals to cells, now recognized not only as a consequence but as a potential driver of disease progression. To elucidate how the ECM influences pathophysiological changes occurring in COPD, in vitro models are needed that incorporate the ECM. ECM hydrogels are a novel experimental tool for incorporating the ECM in experimental setups. We developed an airway wall model by combining lung-derived ECM hydrogels with a co-culture of primary human fibroblasts and epithelial cells at an air–liquid interface. Collagen IV and a mixture of collagen I, fibronectin, and bovine serum albumin were used as basement membrane-mimicking coatings. The model was initially assembled using porcine lung-derived ECM hydrogels and subsequently with COPD and non-COPD human lung-derived ECM hydrogels. The resulting 3D construct exhibited considerable contraction and supported co-culture, resulting in a differentiated epithelial layer. This multi-component 3D model allows the investigation of remodelling mechanisms, exploring ECM involvement in cellular crosstalk, and holds promise as a model for drug discovery studies exploring ECM involvement in cellular interactions.

## 1. Introduction

Chronic respiratory diseases are rapidly becoming a prominent cause of disability and mortality, with chronic obstructive pulmonary disease (COPD) ranking as the world’s third leading cause of death [1]. This condition poses a major public health problem, affecting an estimated global population between 178 million and 328 million individuals [2,3]. COPD develops through an interplay between genetic susceptibility and exposure to damaging environmental stimuli such as cigarette smoke, smoke from indoor cooking, and other harmful particles [4,5].

In general, COPD is a heterogeneous lung condition characterized by chronic respiratory symptoms (dyspnea, cough, expectoration, and exacerbations) arising from abnormalities of the airways (bronchitis, bronchiolitis) and/or alveoli (emphysema), leading to persistent, often progressive, airflow obstruction [6]. Emphysema is the pathological enlargement of the alveoli as a result of chronic inflammation. The presence of an increased number of inflammatory cells leads to destruction of the alveolar walls, and eventually, because of impaired tissue repair, leads to the enlargement of airspaces, defined as emphysema [7,8].

Chronic bronchitis results in pathological alterations around the airways, including increased inflammation, goblet cell metaplasia, mucus hypersecretion, decreased ciliated cell differentiation and airway remodelling, increasing the airway thickness and restricting airflow [8,9]. A major hallmark of bronchitis is the fibrosis of the small airways or bronchioles (diameter < 2 mm), which contributes to air trapping in the alveoli as the small airways narrow, although the precise mechanisms of small-airway fibrosis remain to be fully understood [10].

The small airways are lined with columnar epithelial cells consisting of ciliated cells, goblet cells, and club cells, which all originate from the basal progenitor cells [9]. In COPD, the airway epithelium experiences injury due to repeated exposure to noxious (cigarette smoke) particulate matter and in order to maintain its function, re-epithelialization with differentiated cell types is essential. This epithelial remodelling is impeded in COPD due to the repeated exposure to damaging smoke and the subsequent chronic inflammation [10,11]. The resultant changes in the epithelial layer include goblet cell metaplasia, basal cell hyperplasia, and squamous cell metaplasia [12]. These epithelial cells influence fibroblasts through paracrine signalling, potentially steering them towards a more inflammatory and less regenerative state or promoting their differentiation into myofibroblasts [13,14]. Amidst this cycle of noxious fumes, dysfunctional epithelium, and misguided fibroblasts, a common result is the aberrant remodelling of the extracellular matrix (ECM), which in COPD leads to small-airway fibrosis [15].

The ECM is the three-dimensional network of proteins embedded in a water-retaining gel of polysaccharides that provides structural and biochemical support to cells in all tissues [16]. The ECM consists of a core matrisome of ~300 proteins, glycosaminoglycans (GAGs), proteoglycans (PGs), and structural components. Next to the core matrisome, the ECM also consists of ECM-modifying enzymes such as matrix metalloproteases and lysyl oxidases, growth factors, and other ECM-associated proteins [17]. The ECM can be divided into two specialized structures: the basement membrane (BM) and the interstitial ECM. In the airway wall, the BM is a thin but dense pliable sheet that underlines the basolateral side of the epithelium, whereas the interstitial ECM fills the rest of the space between cells [18]. The structural backbone of the ECM consists of fibrillar proteins such as fibronectin, elastin, and fibrillar collagens [19,20]. The negatively charged polysaccharides give the ECM its water retaining capabilities. The two types of poly(di)saccharides are the GAGs and GAGs covalently attached to a protein core forming PGs [21]. PGs and the attached GAGs form highly extended water-swollen conformations that enable the ECM to withstand high compressive forces [19,21]. During the progression of lung diseases, the ECM undergoes compositional changes which accumulate over time.

The ECM is anything but a passive structure and increasing evidence suggests the ECM plays an active role in disease progression [22,23]. The ECM influences cell behaviour and regulates cell fate through both physical and biochemical interactions [24]. In COPD, fibroblasts of the small airways secrete more collagen 1A1, collagen 3A1, and matrix metalloproteinases, with a resultant increase in ECM deposition [15,25]. At the same time, the persistent inflammation leads to the disruption of normal repair mechanisms, which then together lead to the compositional changes in the small-airway ECM [26]. Due to these changes in composition, the mechanical properties of the ECM change in tandem. The ECM in the emphysematous regions of the lung is less stiff than in healthy regions [27,28]. In contrast, the fibrotic airway has an increased stiffness due to the loss of elastic fibres and accumulation of collagens [22,29]. The stiffness of the ECM is directly related to the biomechanical properties and, with that, the biological cues it gives cells.

In experimental setups, the ECM is generally applied as a coating in semi-3D, i.e., 2.5D models such as air–liquid interface (ALI) cultures and microfluidic chips and is often a combination of 2–3 basement membrane or ECM proteins [30]. Animal models have the ECM exactly in its native conformation but such models often do not accurately mimic the pathophysiological changes in the ECM in chronic human (lung) disease since the disease model is usually induced in a rather short timeframe in contrast to the human disease development and progression which, in the case of chronic lung diseases like COPD, takes years. Hydrogels have been used to mimic the ECM in experimental setups and have been made from both synthetic (polyvinylalcohol, polyacrylic acid, polyethylene glycol) [31,32] as well as natural polymers (collagen, fibrin, alginate, hyaluronic acid) [33,34,35,36]. The synthetic hydrogels often lack biological cues for cells, whereas the natural hydrogels often lack mechanical strength, and neither contain the full composition of the ECM. Organ-derived ECM hydrogels retain most of the ECM constituent proteins of the tissue of origin [37]. The ECM composition is region-specific in organs; this has been shown with both lung and heart ECM, where the specific compartments, airways and alveolar regions for the lung and aorta, mitral valve, and ventricles for the heart, were separated and proteomically analysed [38,39]. For the lung, ECM-derived hydrogels have been made from decellularized porcine and from both healthy and diseased human lung [40,41]. A 3D model of the airway wall that incorporates the ECM of the lung has not yet been developed. In this study, as a proof of concept, we aimed to rebuild airways in 3D using porcine and human lung ECM together with native cellular constituents co-cultured at an air–liquid interface to help elucidate lung pathophysiology; this may be used as a drug discovery platform in the future.

## 2. Materials and Methods

### 2.1. Processing of Lung Tissue and Primary Cells

Lung fibroblasts, epithelial cells, and lung tissues were derived from leftover lung material after lung surgery and transplant procedures. Tissues from human explanted lungs were obtained from tissue remaining after diagnostic procedures from control (non-usable donor lungs and tumour resection material) (*n* = 6) and COPD GOLD IV (*n* = 6) patients undergoing lung transplantation or lung resection in the University Medical Center Groningen. The tumour resection material was obtained as far from the tumour margin as possible and was anatomically normal. The study was conducted in accordance with the Research Code of the University Medical Center Groningen (UMCG), as stated on https://umcgresearch.org/w/research-code-umcg, as well as the national ethical and professional guidelines the Code of Conduct for Health Research (https://www.coreon.org/wp-content/uploads/2023/06/Code-of-Conduct-for-Health-Research-2022.pdf). The use of left-over lung tissue in this study was not subject to the Medical Research Human Subjects Act in the Netherlands, as confirmed by a statement of the Medical Ethical Committee of the University Medical Center Groningen, and therefore, exempt from consent according to national laws (Dutch laws: Medical Treatment Agreement Act (WGBO) art 458/GDPR art 9/UAVG art 24). All donor material and clinical information was deidentified prior to experimental procedures, blinding any identifiable information to the investigators.

The primary epithelial cells were obtained from bronchial brushing through bronchoscopy from healthy donors using a standardized protocol during conscious sedation [42,43,44]. The medical ethics committee of University Medical Center Groningen approved the study (METC 2019/338) and all donors gave informed written consent. The donors (2 female, 3 male) were healthy, nonsmoking volunteers with normal lung function (FEV/FVC > 70%, FEV_1_ > 90% predicted) with an absence of bronchial hyperresponsiveness to methacholine (PC_20_ methacholine > 8 mg/mL). Porcine lungs (~6 months, female, *n* = 2) were purchased from a local slaughterhouse (Kroon Vlees, Groningen, The Netherlands).

### 2.2. Decellularization

The following process was employed for the porcine lungs, as well as the human lungs. The lung was dissected and cartilaginous airways and large blood vessels were removed before cutting the lung into ~1 cm^3^ cubes that were homogenized in a kitchen blender prior to decellularization. The lung homogenate was decellularized as previously described [40,45,46]. In short, the homogenate underwent repeated washing cycles with Milli-Q^®^ water and was centrifuged at 3000× *g* until the supernatant was entirely clear. The sedimented material underwent two consecutive rounds of treatment with various solutions, 0.1% Triton X-100 (Sigma-Aldrich, St. Louis, MO, USA), 2% sodium deoxycholate (Sigma-Aldrich), 1 M NaCl solution, and 30 µg/mL DNase (Sigma-Aldrich) in 1.3 mM MgSO_4_ (Sigma-Aldrich) and 2 mM CaCl_2_ (Sigma-Aldrich) and 10 mM Tris pH8 (Sigma-Aldrich) solution, each for 24 h at 4 °C with constant rotation, except for the DNase treatments, which took place at 37 °C with continuous sideways agitation. A volume ratio of 1:10 was maintained between the tissue homogenate to decellularization/washing solution. Between each treatment, the homogenate was washed three times with Milli-Q^®^ water, with centrifugation at 3000× *g* between washes. The tissue homogenate was sterilized after two cycles of decellularization by adding 0.18% peracetic acid and 4.8% ethanol, with continuous agitation at 4 °C for 24 h. After ECM sterilization, the resulting decellularized ECM was washed three times at 4 °C with sterile Dulbecco’s phosphate-buffered saline (DPBS) and stored in sterile DPBS containing 1% penicillin–streptomycin (Gibco Invitrogen, Carlsbad, CA, USA).

### 2.3. ECM Hydrogel Preparation

The decellularized lung ECM was snap-frozen in liquid nitrogen and freeze-dried with a FreeZone Plus lyophilizer (Labconco, Kansas City, MO, USA), after which it was ground into a powder with an A11 analytical mill (IKA, Staufen, Germany). To solubilize the decellularized ECM, 20 mg/mL of ECM powder was digested with 2 mg/mL of porcine pepsin (Sigma-Aldrich) in 0.01 M HCl under constant agitation at RT for either 48 h (porcine ECM) or 72 h (human ECM). For the solubilization of 10 mg/mL of porcine lung, a 1 mg/mL pepsin solution was used. Digestion was stopped by neutralizing the pH with 0.1 M NaOH and the solution was brought to 1X PBS with the addition of one-tenth volume 10× PBS to produce the lung ECM pre-gel solution, which was stored at 4 °C.

### 2.4. ECM Hydrogel Rheology

The Young’s moduli of the ECM hydrogels were determined using the rheological measurements previously described [40,45,46]. A uniaxial low-load compression tester (LLCT) was used at room temperature to measure hydrogel rheology [47]. The hydrogels were measured with a 2.5 mm ⌀ plunger at three different locations (≥2 mm away from the gel border and ≥2 mm between compression sites). The hydrogels were compressed to 20% of their original thickness (strain ε = 0.2) at a deformation speed of 20%/s (strain rate $\dot{\varepsilon }$ = 0.2 s^−1^). The compression stress was plotted against the strain and a linear increase in stress as a function of strain was observed between a strain of 0.04 and 0.1. The stiffness of the material is essentially described by the Young’s modulus, which was derived from the slope of the line fit to this [30].

### 2.5. Primary Human Fibroblast and Epithelial Cell Isolation and Expansion

The isolation from control donors and consequent expansion was performed as previously described for both the primary lung fibroblasts [48] and the primary bronchial epithelial cells [42]. In short, the fibroblasts were isolated from parenchymal lung tissue by cutting it into small cubes (~1 mm^3^) and placing the cubes in a 12-well culture plate (Corning). The tissue cubes were allowed to adhere for 10 min at 20 °C, after which 1.5 mL of Ham’s F12 medium (BioWhittaker Europe BV, Verviers, Belgium) supplemented with 10% foetal calf serum (FCS; PAA Laboratories, Linz, Austria), l-glutamine (2 mM; BioWhittaker), streptomycin (100 µg/mL; BioWhittaker), and penicillin (100 U/mL; BioWhittaker) was added. The tissue cubes were cultured at 37 °C, 5% CO_2_, and medium was replaced once a week until the primary fibroblast outgrowth was substantial (~5 weeks). Tissue cubes were removed and the fibroblasts were trypsinized (0.05% trypsin/0.02% EDTA; BioWhittaker) and transferred into 25 cm^2^ culture flasks (Corning). When confluent, cell culture media were taken for mycoplasma testing and only used when certified negative. The fibroblasts were frozen and stored in liquid nitrogen.

For the epithelial cells, after bronchoscopy the brush was kept in Hank’s Balanced Salt Solution (HBSS; Gibco) + P/S on ice and centrifuged at 500× *g* for 10 min. The resulting supernatant was removed, and 2 mL of Airway Epithelial Cell Growth Medium (AEGM) (Promocell, Heidelberg, Germany) was added to the pellet. The pellet was resuspended and transferred to a T12.5 cm^2^ culture flask pre-coated with a collagen I–fibronectin–bovine serum albumin solution consisting of 10 μg/mL BSA, 10 μg/mL fibronectin (Sigma Aldrich, St. Louis, MO, USA), and 30 μg/mL collagen (PureCol^®^, Advanced Biomatrix, San Diego, CA, USA) in Eagle’s Minimum Essential Medium (EMEM, Lonza, Walkersville, MD, USA). The cells were then cultured in a 37 °C CO_2_ incubator until reaching 90–95% confluency, which typically took a maximum of 10 days. Upon reaching confluency the cells underwent two washes with HBSS, were detached with 0.5 mL of 0.25% trypsin/EDTA, centrifuged at 500× *g* for 5 min, and then, resuspended in AEGM. They were seeded into pre-coated T25 cm^2^ flasks with 5 mL of AEGM and grown until again reaching 90–95% confluency in a 37 °C, 5% CO_2_ incubator. When confluent, the supernatant was collected for mycoplasma testing, the cells were washed, detached as previously described, and were frozen and stored in liquid nitrogen.

### 2.6. Collagen IV Coating and Live/Dead Staining

To test the collagen IV coating’s feasibility, both a cell viability and a collagen IV immunohistochemical stain were performed. For this, both a collagen I and a porcine lung ECM hydrogel were used. For the collagen I hydrogel, ice-cold rat tail collagen I was mixed with sterile 10× PBS, sterile dH_2_O, and 1M NaOH according to the manufacturer’s protocol [49]. Primary human lung fibroblasts (2.5 × 10^5^/mL) together with media were resuspended with collagen I solution to achieve a final collagen I concentration of 2 mg/mL of collagen I. These human lung fibroblasts were combined with porcine 10 mg/mL ECM hydrogel at the same seeding density. Next, 300 µL of collagen I solution or porcine ECM hydrogel containing cells was pipetted into wells in 48-well plates and allowed to gelate for 30 min at 37 °C, 5% CO_2_. After gelation, 400 µL of cell culture medium was pipetted on top of the collagen gel. The cells were then incubated for 24 h at 37 °C, 5% CO_2_.

After 24 h, a coating of Cellmatrix collagen IV (Nitta Gelatin Inc., Morrisville, NC, USA) was applied on some of the porcine and human ECM hydrogels. The collagen IV (3 mg/mL) was diluted 20× with sterile 0.01 M HCIto a concentration of 150 µg/mL. Then, 100 µL of collagen IV solution was pipetted on to the surface of the hydrogel and left for 1 h, after which 100 µL of fibroblast medium was added and the hydrogel was incubated at 37 °C for 24 h. After that, part of the sample underwent live/dead staining, whereas the rest was used for collagen IV staining to determine the presence of the coating.

The assessment of primary human lung fibroblast viability post-collagen IV coating of the hydrogels involved staining live cells with Calcein AM (Thermo Scientific, Breda, the Netherlands), staining dead cells with propidium iodide (PI; Sigma-Aldrich), and labelling nuclei with 4′,6-diamidino-2-phenylindole (DAPI) (Merck, Darmstadt, Germany), as previously described [46,50]. The hydrogels were washed with HBSS, and subsequently, incubated with serum-free fibroblast medium containing 5 µM Calcein AM, 2 µM PI, and 0.1 μg/mL DAPI for 1 h at 37 °C.

### 2.7. 3D Primary Fibroblast and Epithelial Cell Co-Culture

**Figure 1:**
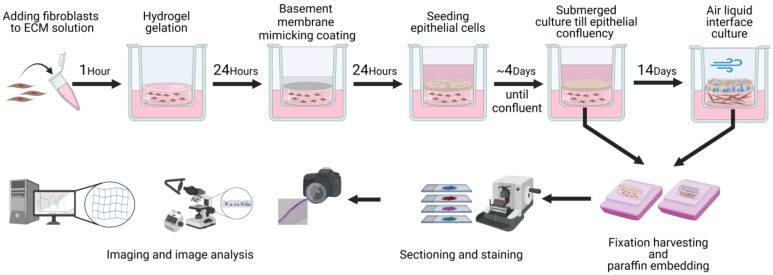
Schematic representation of the model assembly steps. Workflow for the preparation and analysis of the 3D co-culture of primary human fibroblasts and epithelial cells with lung ECM hydrogels. Created with biorender.com.

For the 3D co-culture, THINCERT^®^ cell culture inserts for 24-well plates (Greiner bio-one, Kremsmünster, Austria) were used. Inserts with a 0.4 µm pore size were used for the mono-culture ALI culture, while those with an 8 µm pore size were used for the 3D co-culture with ECM hydrogels. The 3D co-culture setup consisted of primary control human lung fibroblasts within a lung ECM hydrogel (porcine, control human, or COPD) coated with collagen IV, on which primary control human lung epithelial cells were seeded (Figure 1). The epithelial cells and fibroblasts that were seeded together were matched based on sex, age, and smoking status. The fibroblasts were used between passage 3 and 5 and the epithelial cells were used at passage 4. Primary human lung fibroblasts (*n* = 5) were cultured in fibroblast medium consisting of low-glucose Dulbecco’s Modified Eagle Medium (DMEM) (Lonza) supplemented with 10% foetal bovine serum (FBS), 1% penicillin–streptomycin, 1% GlutaMAX (Gibco), and 0.17 mM ascorbic acid. The fibroblasts were washed with HBSS, harvested using 0.25% Trypsin-EDTA (Gibco), and centrifuged at 500× *g* for 5 min. Cells were resuspended in 1 mL of fibroblast medium and counted with a NucleoCounter NC-200™ (Chemometec, Allerod, Denmark). Fibroblasts were mixed into the ECM hydrogels, which were warmed to room temp (~20 °C), at 0.5 × 10^5^ cells/mL. Then, 70 µL of the ECM hydrogel–fibroblast solution was pipetted into a 24-well plate insert and incubated at 37 °C for 1 h, after which 200 µL of fibroblast medium was pipetted on the apical side and 600 µL on the basolateral side. After 24 h, a coating of Cellmatrix collagen IV (Nitta Gelatin Inc., Morrisville, NC, USA) was applied on some of the porcine and human ECM hydrogels. The collagen IV (3 mg/mL) was diluted 20× with sterile 0.01 M HCI to a concentration of 150 µg/mL. Then, 100 µL of collagen IV solution was pipetted on to the surface of the hydrogel and left for 1 h, after which 100 µL of fibroblast medium was added and the hydrogel was incubated at 37 °C for 24 h. After coating, the supernatant was aspirated as well as the basolateral medium. The ECM hydrogels were then washed with HBSS, after which 200 µL of fresh fibroblast medium was added apically and 600 µL basolaterally. The 0.4 µm pore size inserts were coated for 24 h with either 100 µL of collagen IV solution or 100 µL of collagen I–fibronectin–bovine serum albumin solution.

The ALI culture of the 3D co-culture was performed as described before [51,52]. Primary human lung epithelial cells (*n* = 5) were cultured in Bronchial Epithelial Cell Growth Basal Medium (BEBM, Lonza) supplemented with the Bronchial Epithelial SingleQuots™ Kit (Lonza) (BEGM). For the co-culture experiments, ALI culture medium was used. The ALI culture medium was prepared by mixing DMEM (Lonza) and BEBM (Lonza) in a 1:1 ratio supplemented with a set of BEGM SingleQuots (Lonza), 1.5 µg/mL BSA (Sigma-Aldrich, Darmstadt, Germany), and 15 ng/mL retinoic acid (Sigma-Aldrich). The epithelial cells were washed with HBSS, harvested using 0.25% Trypsin-EDTA, and centrifuged at 500× *g* for 5 min. Cells were resuspended in 1 mL of ALI medium and counted with a NucleoCounter NC-200™. The primary human lung epithelial cells from each donor were seeded at a density of 1 × 10^5^ cells/insert to establish a near-confluent layer. When the submerged culture reached confluency (~4 days), half of the inserts were harvested while the other half were air-exposed for 14 days, during which time the basal ALI medium was refreshed each Monday, Wednesday, and Friday.

### 2.8. Histology and Immunohistochemistry

The hydrogels containing embedded fibroblasts, coated with collagen IV, underwent fixation using 4% paraformaldehyde (PFA) overnight at 4 °C. Afterward, they were washed with sterile PBS, dehydrated, embedded in paraffin, and stored. All paraffin-embedded samples were cut into 4 µm thick sections, mounted on slides (VWR, PA, Radnor USA), allowed to dry on a heat plate set at 40 °C for 30 min, and put in an incubator at 37 °C overnight. For histological examination, the slides were deparaffinized using a series of xylene washes followed up with alcohol washes of decreasing concentration (99.6% to 70% ethanol), ending in demi-water [53]. Antigen retrieval was performed by incubating the slides overnight in an 80 °C incubator in Tris/HCl buffer (pH 9). Subsequently, sections were treated for 30 min with hydrogen peroxidase solution (10 µL of 30% H_2_O_2_ per 1 mL of PBS). After washing thrice with PBS, sections were incubated with the primary antibody rabbit-anti-COL4A1 (ab6586, Abcam, Cambridge, UK) at 1:200 in Tris-buffered saline (TBS) with 1% BSA for 120 min at room temperature. After three washes with PBS, the slides were incubated with the secondary antibody, a goat-anti-rabbit multimer horse radish peroxidase (HRP) conjugate (Dako, Santa Clara, CA, USA), at a 1:100 dilution in TBS with 1% BSA + 1% AB human serum for 30 min at room temperature. Lastly, a tertiary antibody was used, a rabbit-anti-goat multimer HRP conjugate (Dako), at a 1:100 dilution in TBS with 1% BSA + 1% AB human serum for 30 min at room temperature. The MUC5AC staining was then visualized with the Ventana^®^ Universal DAB Detection Kit (Roche), and DAB (sigma) was used for tubulin and KRT5. Slides were counterstained with haematoxylin, dehydrated, and covered with Permount^®^ mounting medium.

The 3D co-culture structures of fibroblast–ECM–epithelial cells were fixed in 4% paraformaldehyde (PFA) overnight at 4 °C. Subsequently, they were washed with sterile PBS, embedded in 1% ultrapure agarose (Invitrogen), dehydrated, embedded in paraffin, and stored. All paraffin-embedded samples were cut into 3 µm thick sections, mounted on slides, and dried on a heat plate set at 60 °C for 30 min, followed by overnight incubation at 37 °C. For histological examination, the slides were deparaffinized as described in Section 2.6. The sections were then subjected to staining procedures: Haematoxylin and eosin (H&E) [54] for a general overview of the organization, shape, and structure of various cells and ECM in the co-culture samples; Alcian blue (CLIN-TECH, Guildford, UK) with a nuclear fast red counterstain (Merck) [45] to identify mucus-positive cells; and Picrosirius red (PSR; Sigma-Aldrich) [46] to visualize collagens. After staining, slides were covered with Permount™ mounting medium (Fisher Chemical™, Waltham, MA, USA) for preservation.

For identification of different human epithelial cells, we performed immunohistochemical staining targeting mucin-5AC (MUC5AC) (goblet cells), tubulin (ciliated cells), and keratin 5 (KRT5) (basal cells). For staining, slides with 3 µm sections were deparaffinized and subjected to antigen retrieval by incubating at 100 °C for 15 min in the microwave in Tris-HCI buffer (pH 9) for MUC5AC or citrate buffer (pH 6) for tubulin and KRT5. Subsequently, sections were incubated for 30 min in hydrogen peroxidase solution (10 µL of H_2_O_2_ per 1 mL of PBS). After washing with PBS, sections were either incubated with 1:1600 of mouse-anti-MUC5AC (ab3649, Abcam, Cambridge, UK), 1:10,000 of mouse-anti-acetylated tubulin (t7451, Sigma-Aldrich), or 1:3000 of chicken-anti-KRT5 (sig-3475, Biolegend, San Diego, CA, USA) for 60 min at room temperature. After three PBS washes, sections were incubated with their specific secondary antibodies. For MUC5AC, the secondary antibody used was undiluted rabbit-anti-mouse multimer HRP conjugate (Dako, Santa Clara, CA, USA). For tubulin, a 1:100 rabbit-anti-mouse HRP conjugate (Dako) was applied, and for KRT5, a 1:100 dilution of rabbit-anti-chicken biotin (Jackson Immunoresearch, Cambridge, UK) was used, followed by incubation with 1:300 streptavidin HRP (Dako) for 30 min. Visualization of staining was performed using the Ventana^®^ Universal DAB Detection Kit (Roche, Rotkreuz, Switzerland). Slides were counterstained with haematoxylin and covered with Permount^®^ mounting medium for preservation.

### 2.9. Imaging and Image Analysis

Fluorescent images of the live/dead staining were captured using an EVOS cell imaging system (Thermo Scientific) with GFP (509 nm), Texas Red (615 nm), and DAPI (460 nm) channels. All colorimetrically stained sections were scanned with a NanoZoomer slide scanner (Hamamatsu Photonics, Herrsching, Germany) at 40× magnification (20,480 pixels width, 14,080 pixels height, 0.227 microns per pixel). The scanned images from the stained ALI culture or 3D co-culture membranes were quantified by measuring the length of the membrane and counting the number of positive cells for each staining. These data were expressed as number of positive cells per µm membrane or percentage positive cells of the total cells. The counting was performed by two independent observers.

**Figure 2:**
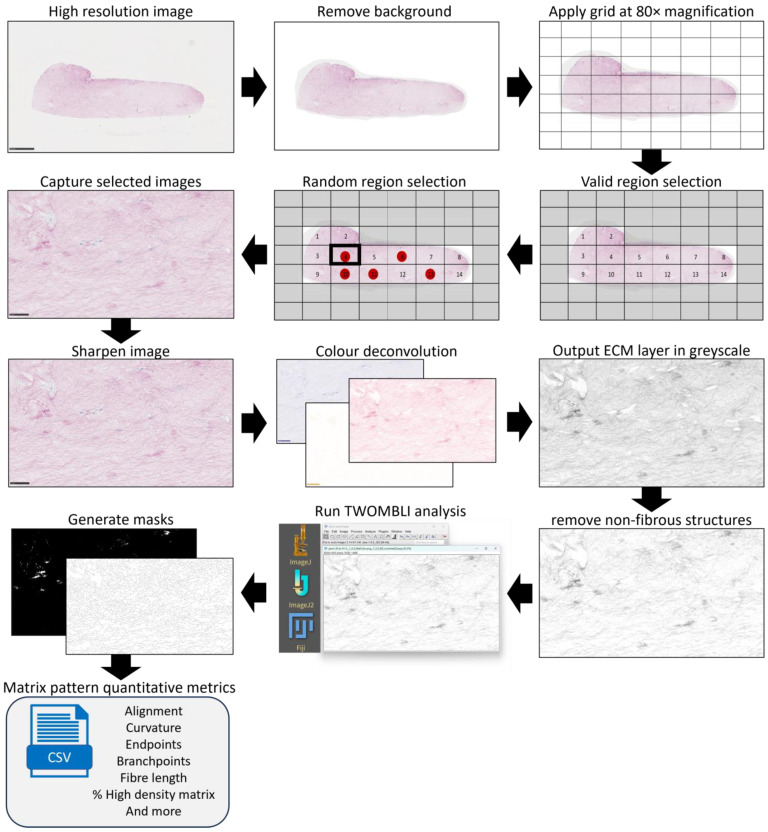
TWOMBLI analysis image preparation. Workflow for the preparation of Picrosirius red-stained (PSR) high-resolution images for TWOMBLI analysis of extracellular matrix organization. Eligible regions (those containing ECM hydrogel with no major defects and abnormalities) were identified, and then, 5 regions of interest were selected through a random number generator. Images were taken at 80× magnification of the selected regions and sharpened. These images were then processed using Fiji ImageJ to separate the specific signal for the red (PSR) signal. Lastly, the TWOMBLI macro generated high-density matrix and fibre masks from the processed images, from which the quantitative metrics were generated.

PSR-stained porcine and human lung ECM hydrogel co-culture high-resolution images were captured using the Hamamatsu slide scanner as described above. The workflow for image selection and preparation for TWOMBLI analysis is presented in Figure 2. In short, the image was cropped to the area of interest and cleaned to only contain the hydrogel and remove irregularities using Photoshop 25.1.0 release (Adobe, San Jose, CA, USA). A grid was then applied over the image with a square size of 165 µm by 295 µm (L × W) and the regions containing ECM hydrogel and no major abnormalities were numbered. Using a random number generator, 5 valid regions were selected per image and captured (80× magnification) from the high-resolution image using the NDP.view2 software (Hamamatsu Photonics). The regional images were sharpened using Photoshop with the unsharp mask (160% with a radius of 3.0 pixels). The red colour of the sharpened 80× images was isolated using the Fiji ImageJ (version 2.14.0) [55] plugin colour deconvolution; the ECM layer output was isolated in greyscale, from which undefined structures were removed [56]. The TWOMBLI macro [57] for Fiji ImageJ was used to generate fibre and high-density masks, and then, to assess percentage high-density matrix (HDM), alignment, fibre length, end points, branching points, and curvature of the fibres, as previously described [45,46,57].

**Figure 3:**
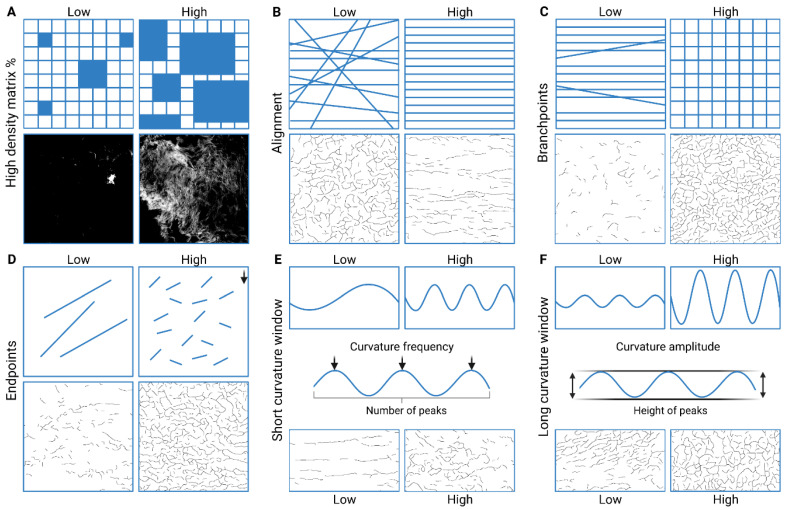
TWOMBLI analysis matrix metrics. Images show schematic examples of matrix metrics together with examples of TWOMBLI-generated masks from the Picrosirius red-stained ECM hydrogels. (**A**) Percentage of high-density matrix indicating accumulated matrix. (**B**) Alignment of matrix fibres. (**C**) Branchpoints: the number of intersections of mask fibres in the image. (**D**) Endpoints: number of fibre end points. (**E**) Short-curvature windows: describing the frequency of the peaks in the fibre curvature. (**F**) Long-curvature windows: describing the amplitude of the peaks in the fibre curvature. Adapted from [57] (published under CC-BY 4.0). Created with BioRender.com.

The matrix metrics measured in this study describe the organization of the ECM, with each metric highlighting a specific factor in the topology (Figure 3). HDM indicates the percentage of accumulated ECM in a region, alignment shows if the ECM fibres are arranged in the same direction, fibre length shows the average length of fibres, branchpoints shows how much the ECM fibres branch, and endpoints gives the number of fibre ends. The average fibre length is calculated by dividing total fibre length by 0.5× (endpoints + branchpoints), assuming that one of the fibres involved at a branch begins at the branch point. The curvature indicates the change in angle moving incrementally along individual fibres. Curvature is divided into two parameters to describe the curves: short-curvature windows (10–50 degrees), indicating the frequency or periodicity of the curvature; and long-curvature windows (60–100 degrees), reflecting the amplitude of the curves.

### 2.10. Statistical Analyses

Statistical analyses were performed using the PRISM v10.1.2 software (GraphPad Prism, San Diego, CA, USA). Data are presented as individual values as well as the mean with standard error of the mean (SEM). Five regions of interest per sample were used for the TWOMBLI metrics (% high-density matrix, alignment, hyphal growth unit, branchpoints, and endpoints), the raw data of which are presented in Supplementary Figures S1–S4. The TWOMBLI metrics data were analysed for normality using Shapiro–Wilk and Q-Q plots (Supplementary Figures S5–S7); non-normal data were transformed. When the transformed data were not normally distributed, the untransformed data were presented. Linear mixed models were also used to investigate differences in TWOMBLI metrics between ALI and submerged cultured 3D models for both porcine and human lung ECM hydrogel models. Data were analysed using IBM SPSS version 29.0.1.0(171). Scatter and forest plots were created in GraphPad Prism version v10.1.2. Differences were considered significant when *p* < 0.05.

## 3. Results

### 3.1. Collagen IV Coating of ECM Hydrogel Containing Human Lung Fibroblasts

**Figure 4:**
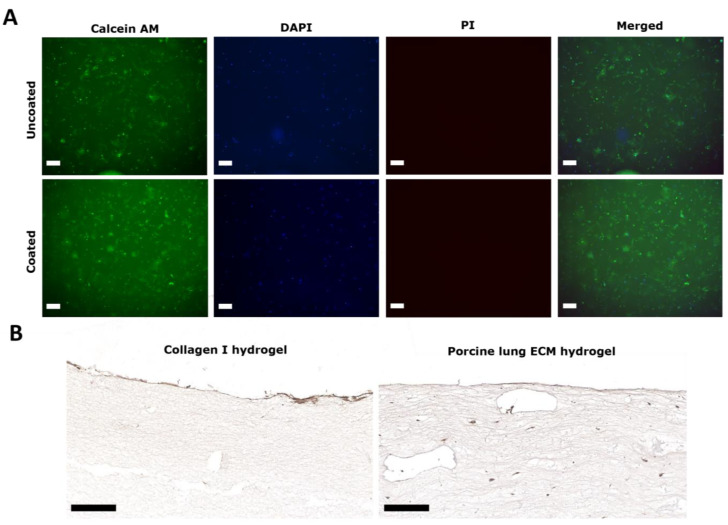
Collagen IV coating of collagen I and porcine lung ECM hydrogels on cell viability. (**A**) Collagen I-embedded fibroblast viability 1 day after collagen IV staining procedure. Calcein AM (green) staining of live fibroblasts; propidium iodine (PI; red) staining of dead cells; DAPI (blue) indicating nuclei; and a brightfield overview. The scale bars represent 200 μm. (**B**) Immunohistochemical staining of collagen IV on top of fibroblast-seeded collagen I and 10 mg/mL porcine lung ECM hydrogels 24 h after coating. The scale bars represent 100 μm. Results are representative for all experiments (*n* = 2).

As a first step to modelling the airway wall, we examined the influence of the basement membrane coating on the viability of the fibroblasts. One day after the collagen IV coating procedure, the fibroblasts remained viable, as evident from the positive Calcein AM staining and the absence of PI staining (Figure 4A). The brightfield images also showed no discernible differences in morphology in the presence or absence of the collagen IV coating. The collagen IV coated both the collagen I hydrogel and the porcine hydrogels, as visualized by the collagen IV immunostaining (Figure 4B).

### 3.2. Porcine ECM Stiffness Depends on Concentration

**Figure 5:**
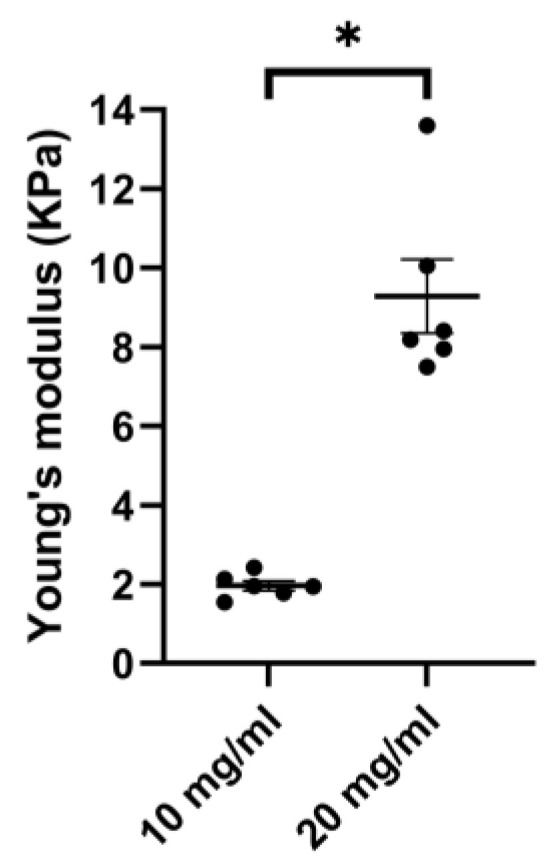
Porcine lung ECM hydrogel’s Young’s modulus. Comparison of stiffness of porcine lung ECM hydrogels prepared either at a concentration of 10 mg/mL or 20 mg/mL. For each hydrogel condition, 3 replicate gels were made and measured individually on 2 separate occasions. Mann–Whitney U test was used to compare the two concentrations; * *p* < 0.005.

To explore the possibility of mimicking the airway wall stiffness as seen in health and disease, we examined the stiffness of porcine ECM hydrogels of 10 and 20 mg/mL. Both the 10 mg/mL and 20 mg/mL porcine lung hydrogels formed stable gels upon gelation at 37 °C. When increasing the concentration of the porcine ECM hydrogel from 10 mg/mL to 20 mg/mL the stiffness increased. The average 20 mg/mL hydrogel stiffness was higher (9.28 ± 2.29 kPa) than that of the 10 mg/mL hydrogel (1.96 ± 0.29 kPa) (Figure 5).

### 3.3. Hydrogel Co-Culture Contraction Starts after Epithelial Cell Seeding

**Figure 6:**
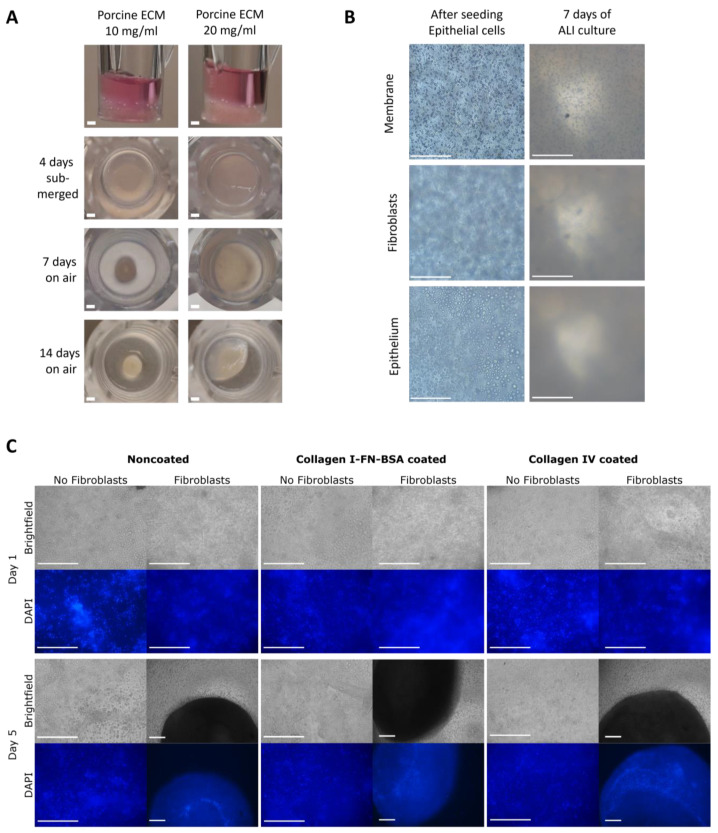
Model assembly with porcine lung ECM hydrogel and its contraction over time. (**A**) Macroscopic view of the 10 and 20 mg/mL porcine lung ECM hydrogel 3D co-culture model: side view directly after full assembly; top view after 4 days submerged culture; 7-day ALI culture; and 14-day ALI culture. (**B**) Microscopic (20×) images after initial seeding of epithelial cells on and fibroblast seeding in ECM hydrogel showing reduced visibility after hydrogel contraction. (**C**) Brightfield and fluorescent microscopic images (10×): images 1 and 5 days after epithelial cell seeding on top of fibroblast-containing or empty 10 mg/mL porcine lung ECM hydrogels. Cells are stained with DAPI to visualize nuclei. ECM: extracellular matrix, ALI: air–liquid interface, FN: fibronectin, BSA: bovine serum albumin, DAPI: 4′,6-diamidino-2-phenylindole.

The interplay between epithelial cells and fibroblasts in the gel was examined over time during the initial stages of the model assembly. When assembled, the fibroblast and epithelial co-culture model retained its shape with a slightly concave surface (first panel of Figure 6A). The apical side of the ECM co-culture model contracted visibly during the 4 days of submerged culture, as indicated with the back arrows in the second panel of Figure 6A. In the first 7 days of the ALI culture, the entire model contracted further, with the 10 mg/mL porcine lung hydrogel showing a higher degree of contraction than that of the 20 mg/mL hydrogel. No further contraction of the model was observed during the last 7 days of the 14-day ALI culture. The initial visibility via brightfield microscopy was clear, whereas this clarity rapidly declined in the contracted state of the model (Figure 6B). Apical side contraction after the submerged culture occurred irrespective of the basement membrane coating strategy, as seen in the placement of the cell in the fluorescent images (Figure 6C). The combination of fibroblasts and epithelial cells contracted the ECM hydrogel to a larger extent than epithelial cells alone, the visualization of which in severely contracted hydrogels was made possible through DAPI nuclear staining (Figure 6C).

### 3.4. Variation of Hydrogel Co-Culture Contraction

**Figure 7:**
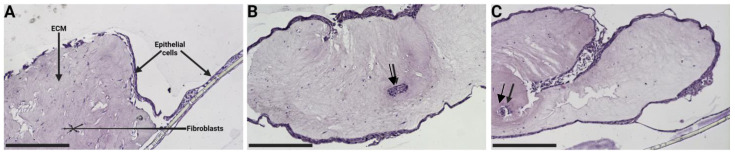
Different porcine lung ECM hydrogel contraction outcomes. Haematoxylin and eosin-stained porcine lung ECM hydrogel co-culture model after 14 days of air–liquid interface culture. (**A**) Epithelial confluency spanning from the hydrogel across the membrane. (**B**) Epithelial confluence covering the entire contracted ECM and small enveloped accumulated cell pockets (arrow). (**C**) Folding of the entire hydrogel during contraction. Scale bars are 500 µm. ECM: extracellular matrix.

The contraction of the 3D ECM hydrogel co-culture model, seen in Figure 6A,C, was further visualized using histology (Figure 7). This contraction exposed the insert membrane, allowing the epithelial cells to grow onto it and spread across until reaching the edge of the transwell (Figure 7A). The epithelial cells were observed to have passed through and be growing on the underside of the insert membrane (Figure 7A). Once the 3D model had fully detached from the transwell membrane, the epithelial cells completely enveloped the ECM hydrogel (Figure 7B). Occasionally, a small group of enveloped cells could be observed within the ECM hydrogel (indicated by the black arrow in Figure 7B). During the contraction process, there were rare instances where the 3D model folded into itself due to the forces exerted by the cells on the ECM hydrogel (Figure 7C). 

### 3.5. Epithelial Cell Differentiation Irrespective of Coating Strategy

**Figure 8:**
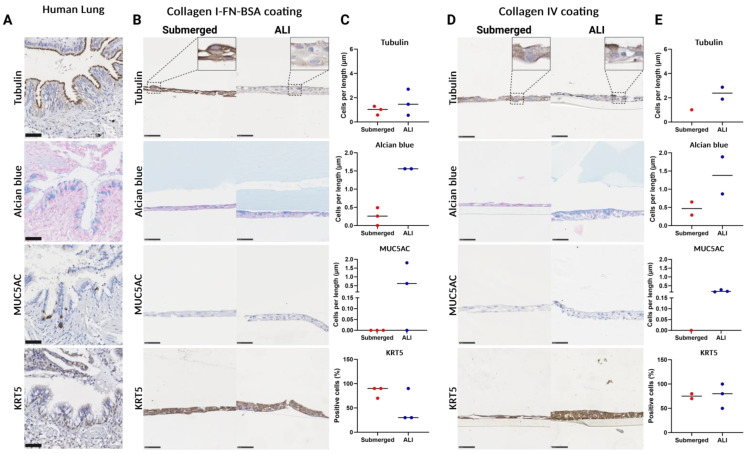
Histological analysis of primary airway epithelial cell differentiation prior to and after air–liquid interface (ALI) culture. Tubulin immunostaining (ciliated cells), Alcian blue staining (goblet cells), MUC5AC immunostaining (club cells), and KRT5 immunostaining (basal cells). Submerged culture was fixed after epithelial cells reached confluence, whereas ALI culture exposed the submerged cultured cells to air for 14 days before fixation. (**A**) Intact control human lung. (**B**) Representative images of sections (40× magnification) of submerged- and ALI-cultured airway epithelial cells on top of a collagen I–fibronectin–bovine serum albumin-coated membrane. (**C**). Quantified differentiated epithelial cells on membranes coated with collagen I-FN-BSA. (**D**). Representative images of sections (40× magnification) of submerged- and ALI-cultured airway epithelial cells on top of a collagen IV-coated membrane. (**E**) Quantified differentiated epithelial cells on membranes coated with collagen IV. Scale bars are 50 µm. All data are shown as means with all available data plotted individually (*n* = 5). FN: fibronectin, BSA: bovine serum albumin, ALI: air–liquid interface, MUC5AC: mucin-5AC, KRT5: keratin 5. Made with Biorender.com.

A regular ALI culture was performed to evaluate the effect of the BM coating on epithelial differentiation and to serve as a control. The differentiated epithelial cells of interest, as shown in the airways of human lung, are as follows: ciliated cells (tubulin), goblet cells (Alcian blue), club cells (MUC5AC), and basal cells (KRT5) (Figure 8A). The ALI culture was compared to the submerged culture and, due to the irregularity in the number of successfully retrieved membranes, no statistical tests were performed. The collagen I-FN-BSA-coated membrane supported a multicellular epithelial layer both before and after ALI culture, with the latter containing differentiated epithelial cells (Figure 8B). After ALI culture, there was a positive trend towards an increase in the number of tubulin-positive cells, Alcian blue-positive cells and MUC5AC-positive cells compared to the submerged cultured epithelial cells (Figure 8C). ALI-cultured epithelial cells on the collagen I–fibronectin–BSA coating showed a negative trend in KRT5-positive cell proportions compared to submerged culture, which could mean more differentiation at the expense of basal cells. The collagen IV-coated membranes similarly showed a multicellular epithelial layer after ALI culture compared to submerged culture similar to the collagen I-fibronectin-BSA coating (Figure 8D). The ALI-cultured epithelial cells showed an increasing trend in numbers of all differentiated cell types but no difference in KRT5-positive basal cell proportions (Figure 8E).

### 3.6. Porcine Lung ECM Hydrogel Concentration Does Not Affect Epithelial Differentiation

**Figure 9:**
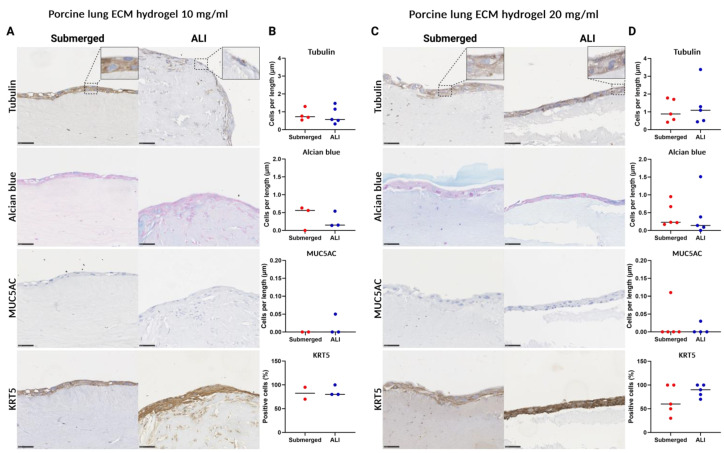
Histological analysis of primary airway epithelial, fibroblast, and porcine lung ECM co-culture model of cell differentiation prior to and after air–liquid interface (ALI) culture. Tubulin immunostaining (ciliated cells), Alcian blue staining (goblet cells), MUC5AC immunostaining (club cells), and KRT5 immunostaining (basal cells). Submerged culture was fixed after epithelial cells reached confluence, whereas ALI culture exposed the submerged cultured cells to air for 14 days before fixation. (**A**) Representative images of sections (40× magnification) of submerged- and ALI-cultured porcine ECM (conc. 10 mg/mL) epithelial cell fibroblast co-culture model. (**B**). Quantified differentiated epithelial cells on membranes coated with collagen I-FN-BSA. (**C**) Representative images of sections (40× magnification) of porcine ECM (conc. 20 mg/mL) epithelial cell fibroblast co-culture model. (**D**) Quantified differentiated epithelial cells on membranes coated with collagen IV. Scale bars are 50 µm. All data are shown as means with all available data plotted individually (*n* = 1–5). ECM: extracellular matrix, ALI: air–liquid interface, MUC5AC: mucin-5AC, KRT5: keratin 5. Made with Biorender.com.

In assembling the 3D ECM hydrogel co-culture model from porcine lung ECM hydrogels, concentrations of both 10 mg/mL and 20 mg/mL were used, each coated with collagen IV. Due to irregularities in the number of successfully retrieved 3D co-culture models, no statistical tests were performed. The 10 mg/mL porcine lung ECM hydrogel 3D co-culture showed a thin multicellular epithelial layer atop the submerged cultured model, with little increase in thickness after ALI culture (Figure 9A). The 10 mg/mL hydrogel showed no change in epithelial cell differentiation after ALI culture compared to the submerged culture (Figure 9B). The representative images for the 20 mg/mL 3D porcine lung ECM hydrogel co-culture showed a similar picture, with a multicellular epithelial layer evident in both the submerged culture and after ALI culture, exhibiting similar thickness (Figure 9C). No changes were noted in the numbers of cells stained for tubulin-, Alcian blue-, and MUC5AC-positive cells per length of the hydrogel after ALI culture compared to submerged culture (Figure 9D). The submerged 20 mg/mL 3D porcine lung ECM hydrogel co-culture showed a lower trend in the proportion of KRT5-positive cells, compared to the ALI culture.

### 3.7. Human ECM Hydrogel Co-Culture Contraction

**Figure 10:**
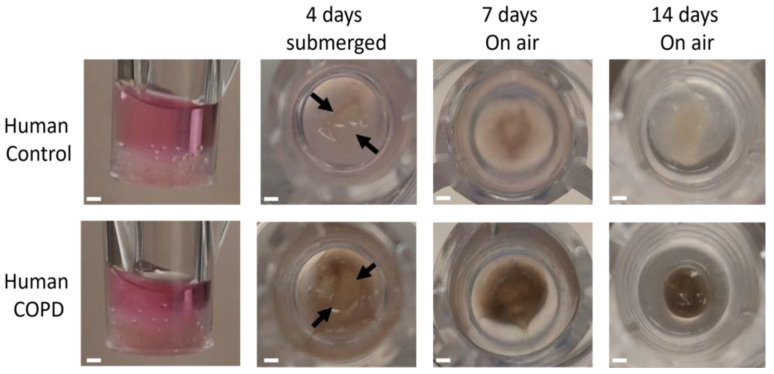
Model assembly with human lung ECM hydrogel and its contraction over time. Macroscopic view of the control and COPD human lung-derived ECM hydrogel 3D co-culture model: side view directly after full assembly; top view after 4 days submerged culture; 7-day ALI culture; and 14-day ALI culture.

After generating the model with the porcine lung ECM hydrogel, we aimed to demonstrate the feasibility of using human lung ECM hydrogels in the model assembly. Similar to the porcine ECM hydrogel 3D co-cultures, the 3D co-cultures made with both control and COPD human lung-derived ECM hydrogel exhibited hydrogel contraction. When assembled, the 3D human lung co-culture model retained its shape, with a slight concave surface (first column of Figure 10). Notably, the COPD-derived human hydrogel appeared darker and brownish due to the smoke particulates that remained during the decellularization and solubilization process (Figure 10). The top layer of the human lung-derived co-culture models contracted during the initial 4 days of submerged culture (indicated by black arrows in the second column of Figure 10). Within the first 7 days of ALI culture, further contraction was observed, with the COPD-derived human lung hydrogel showing a larger degree of contraction compared to the control human lung hydrogel. Notably, there was no additional contraction during the last 7 days of the 14-day ALI culture period regarding the control ECM but the COPD ECM hydrogel continued to contract.

### 3.8. 3D ECM Hydrogel Co-Culture Is Possible with Both Control and COPD Lung ECM Hydrogels

**Figure 11:**
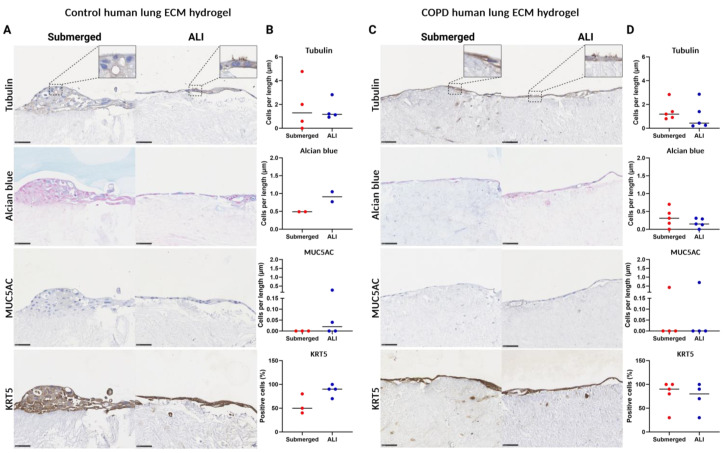
Histological analysis of primary airway epithelial, fibroblast, and human lung ECM (HECM) co-culture model of cell differentiation prior to and after air–liquid interface (ALI) culture. Tubulin immunostaining (ciliated cells), Alcian blue staining (goblet cells), MUC5AC immunostaining (club cells), and KRT5 immunostaining (basal cells). Submerged culture was fixed after epithelial cells reached confluence, whereas ALI culture exposed the submerged-cultured cells to air for 14 days before fixation. (**A**) Representative images of sections (40× magnification) of submerged- and ALI-cultured control human lung ECM hydrogel epithelial cell fibroblast co-culture model. (**B**). Quantified differentiated epithelial cells on membranes coated with collagen I-FN-BSA. (**C**). Representative images of sections (40× magnification) of submerged- and ALI-cultured COPD human lung ECM hydrogel epithelial cell fibroblast co-culture model. (**D**) Quantified differentiated epithelial cells on membranes coated with collagen IV. Scale bars are 50 µm. All data are shown as means with all available data plotted individually (*n* = 5). ALI: air–liquid interface, MUC5AC: mucin-5AC, KRT5: keratin 5. Made with Biorender.com.

In order to assess the feasibility of using healthy and diseased human lung ECM hydrogels in the model assembly, we evaluated the epithelial differentiation as for the model generated from porcine ECM. In assembling the 3D ECM hydrogel co-culture model from human lung ECM hydrogels, both control and COPD lung-derived ECM hydrogels were used, each coated with collagen IV. Also, here, no statistical tests were possible due to variation in the models. A confluent layer of epithelial cells was present in both the control and the COPD ECM hydrogel co-culture model (Figure 11). In the control human lung ECM hydrogel co-culture model, a multicellular layer was initially observed in the submerged culture, which became less pronounced after ALI culture, showing larger areas with a monolayer of epithelial cells (Figure 11A). Despite the less pronounced multilayered structure post-ALI, compared to porcine ALI, differentiated epithelial cells were still observed. There was an increase in Alcian blue-positive mucus-producing cells, as well as MUC5AC- and KRT5-positive cell proportions, with the ALI culture compared to submerged culture (Figure 11B). On the human COPD lung ECM hydrogels, the epithelial cells grew into a confluent layer that persisted after ALI culture, sometimes developing into a multicellular layer with differentiated epithelial cells (Figure 11C). The percentage of differentiated cells remained similar between submerged- and ALI-cultured cells on the COPD hydrogels (Figure 11D).

### 3.9. Changes in the Porcine ECM Organization after ALI Culture

**Figure 12:**
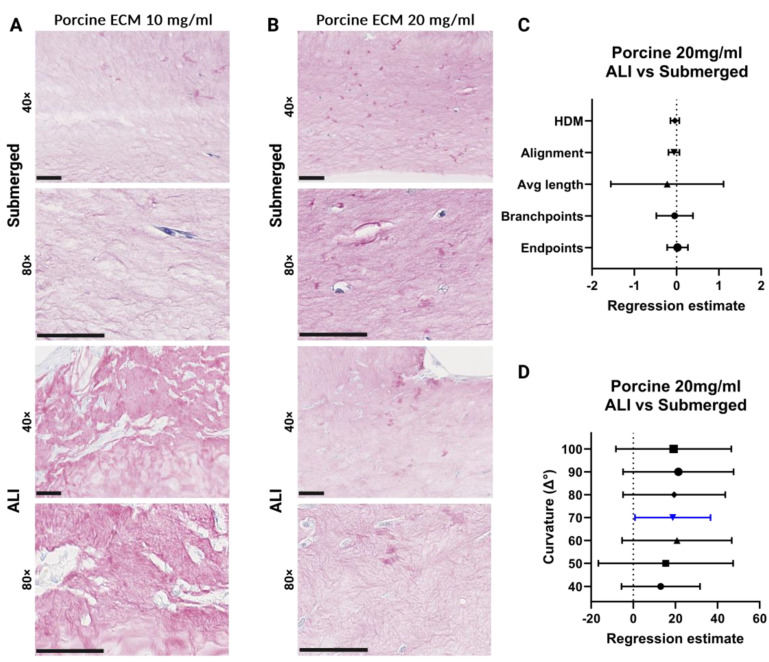
Analysis of porcine lung-derived hydrogel extracellular matrix organization. (**A**) Picrosirius red (PSR)-stained 10 mg/mL porcine lung ECM hydrogel co-cultures. (**B**) PSR-stained images of 20 mg/mL porcine lung ECM hydrogel co-cultures: cultured submerged and at ALI. (**C**) Forest plot of regression estimates for the TWOMBLI metrics percentage high-density matrix (HDM), fibre alignment, average fibre length, fibre branchpoints, and endpoints. (**D**) Forest plot of regression estimates for curvature windows with respect to the periodicity of peaks and peak height of matrix fibres. The dotted line shows the intrinsic difference between ALI and submerged culture. From each hydrogel image, 5 regions of interest were analysed. The applied statistical test was a mixed-model analysis where for each matrix pattern metric the regression estimates (±95% CI) were obtained following linear regression, where a *p* < 0.05 was considered significant. Blue-coloured data points highlight significant differences. Scale bars: 50 μm. ECM: extracellular matrix, ALI: air–liquid interface, HDM: high-density matrix. Made with Biorender.com.

With the 3D model fully assembled, we aimed to characterize the rearrangement of the ECM structure by the cells, as indicated by the hydrogel contraction observed during culture. Representative images of PSR-stained porcine ECM hydrogels showed little difference in staining intensity between 10 and 20 mg/mL in the submerged culture (Figure 12A,C). Both concentrations of hydrogels exhibited higher PSR staining intensity after ALI culture. The 10 mg/mL porcine hydrogel contracted substantially and was therefore difficult to process, section, and stain. Due to the low amount of successfully retrieved 10 mg/mL porcine lung hydrogel co-culture, no statistical tests were performed for this group, so only the 20 mg/mL porcine hydrogel was analysed. Regression estimates (±95% confidence interval [CI]) for the 20 mg/mL porcine lung 3D co-culture showed no difference after ALI culture for percentage high-density matrix (HDM), fibre alignment, average fibre length, fibre branchpoints, and endpoints (Figure 12C). An overall positive trend was observed with increased values in the ALI-cultured 20 mg/mL porcine ECM hydrogel compared to submerged cultures for all curvature windows (Figure 12D).

### 3.10. Changes in the Human ECM Organization after ALI Culture

**Figure 13:**
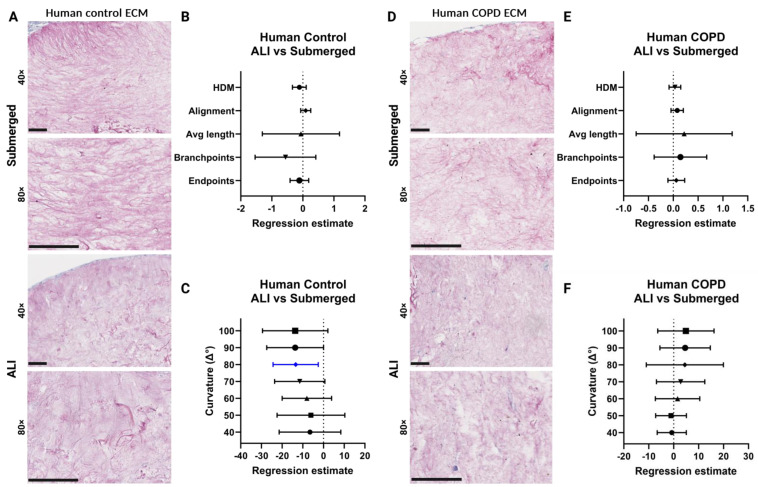
Analysis of human lung-derived hydrogel extracellular matrix organization. (**A**,**D**) Representative images of Picrosirius red (PSR) staining on control and COPD human lung ECM hydrogel co-cultures. (**B**,**E**) Forest plot of regression estimates for the TWOMBLI metrics percentage high-density matrix (HDM), fibre alignment, average fibre length, fibre branchpoints, and endpoints of ALI-cultured compared to submerged for control and COPD lung ECM hydrogel respectively. (**C**,**F**) Forest plot of regression estimates for curvature windows with respect to the periodicity of peaks and peak height of matrix fibres of ALI-cultured compared to submerged for control and COPD lung ECM hydrogel, respectively. The dotted line shows the intrinsic difference between ALI and submerged culture. From each hydrogel image, 5 regions of interest were analysed. The applied statistical test was a mixed-model analysis where for each matrix pattern metric the regression estimates (±95% CI) were obtained following linear regression, where *p* < 0.05 was considered significant. Blue-coloured data points highlight significant differences. Scale bars: 50 μm. ECM: extracellular matrix, ALI: air–liquid interface, HDM: high-density matrix. Made with Biorender.com.

For human lung ECM hydrogels, the differences in ECM organization were examined between healthy and diseased lung ECM as a source for the 3D model. Representative images of the PSR-stained human ECM hydrogels showed little difference in the staining intensity between the control and COPD in the submerged cultures (Figure 13A,D). Also, when comparing the submerged cultures with ALI cultures, representative images of the PSR-stained human ECM hydrogel co-culture model showed little difference in the staining intensity for the control (Figure 13A). This similarity was further supported by regression estimates (±95% CI) of the matrix metrics, revealing no differences between ALI and submerged cultures (Figure 13B). Regression estimates for the curvature window metrics showed an overall negative trend and were decreased in the ALI-cultured control human ECM hydrogel compared to submerged (Figure 13C). For the COPD human lung ECM hydrogel co-culture model, the representative images also showed little difference in the staining intensity between the submerged and ALI cultures (Figure 13D). This is supported by the forest plot of the regression estimates (±95% CI) of the matrix metrics which showed no difference between ALI and submerged culture for COPD human lung ECM hydrogels (Figure 13E). The regression estimates for the curvature windows showed an overall positive trend and were increased in ALI-cultured COPD human ECM hydrogel compared to submerged (Figure 13C). With the exception of curvature, the ECM of control and COPD human lung ECM hydrogels did not undergo major changes with regards to the organization of the ECM fibres after ALI. After ALI culture, the COPD and control hydrogels showed a different trend, where in the control the average length, branchpoints, and endpoint showed a decreasing trend, whereas COPD showed a positive trend for the same metrics. The control hydrogel showed a decreasing trend for all curvature windows, the exact opposite trend to COPD, which had an increasing trend for all. The control hydrogels showed a decreasing trend, which was mainly present in the large-curvature windows and the COPD hydrogels showed an increasing trend, mainly present in the large-curvature windows.

## 4. Discussion

In this study, we developed a novel co-culture model involving primary human lung fibroblasts and bronchial epithelial cells, integrating the lung ECM. Remarkably, this model proved successful across various concentrations of porcine lung ECM, as well as control and COPD human lung ECM. Through ALI culture, the epithelial cells atop this model matured into a multicellular differentiated layer. This innovative model provides a platform to explore both direct and indirect interactions between fibroblasts, epithelial cells, and the ECM. The possibilities of implementing both control and diseased lung ECM, alongside the flexibility to vary ECM concentrations, allows for simulating the ECM accumulation observed in the small airways in COPD. This, in turn, facilitates the study of mechanisms driving airway remodelling in vitro.

The ALI culture method is the gold standard technique for culturing human airway epithelial cells, mimicking the pseudostratified mucociliary phenotype, typically seen in vivo [58]. Coating the supporting membrane for ALI, as used in this study, is not a new concept. In the 1980s, it was recognized that a BM mimic would aid epithelial cell differentiation in ALI culture [59]. Since then, many BM analogues have been developed, such as porous polymeric membranes, electrospun membranes, or basement membrane-derived hydrogels such as Matrigel [60]. In ALI, the porous membrane is usually coated with a collagen solution, often collagen I, sometimes together with other ECM proteins such as fibronectin [61]. The BM, however, consists mostly of a network of collagen IV and laminin isoforms linked together with proteoglycans such as nidogen, perlecan, and agrin [62]. We therefore started with a collagen IV coating in our model, which demonstrated performance akin to the standard collagen I coating in our experiments. This collagen IV coating was applied to all ECM hydrogels in the co-culture setup.

Alterations in the basement membrane composition occur in obstructive airway diseases [63]. In COPD, the basement membrane is thicker than in non-diseased lungs. Moreover, at sites of epithelial damage, there is an increase in both collagen IV and laminin β2 [63,64,65]. These observations suggest that collagen IV serves as a suitable BM analogue in our model setup. It is plausible that the addition of other BM components could exert different influences on epithelial differentiation.

We observed differentiated epithelial cells in both porcine and human ECM hydrogel co-culture models, albeit to a lesser extent compared to tissue sections from native human lung small airways. Numerous ALI protocols exist with varying air exposure durations, lasting anywhere from 14 to 33 days [61,66]. It is possible that the epithelial layer atop the ECM hydrogels might have been in the early stages of differentiation and could potentially benefit from longer ALI cultures. This avenue should be explored in future research to optimize and enhance the differentiation process.

Tissue stiffness regulates cell behaviour, and if we expand on tissue mechanics, it would be more accurate to say tissue viscoelasticity regulates cell fate [67,68]. Even though the difference between the Young’s moduli of the two porcine ECM hydrogels was relatively minor, it is possible that this difference affected the cells cultured atop and within these hydrogels. The human ECM hydrogels displayed similar viscoelastic properties and stiffness as their intact lung counterparts [40]. We did not measure the stiffness of intact porcine lung; however, the stiffness of the 10 mg/mL porcine lung ECM hydrogel (1.96 ± 0.29 kPa) was similar to that of the control human lung ECM hydrogel (1.1 ± 0.2 kPa) and intact human lung (3.7 ± 1.3 kPa) [40]. The 20 mg/mL porcine hydrogel was stiffer than any other hydrogel used, explaining the lower contraction and easier handling. Importantly, one cannot increase the ECM concentration of the hydrogel without also changing other parameters such as stiffness, pore size, ECM fibre architecture, and topology [45]. The concept of cells contracting hydrogels is anything but new and is what is measured with contraction assays to evaluate processes such as myofibroblast conversion and activation [69]. The contraction seen in these collagen gels is most likely not a contraction of the cells themselves but a rearrangement of the ECM structure [70]. An approach to negate the ECM hydrogel contraction would be to mechanically or physically link the ECM hydrogel to the culture transwell insert membrane or wall. A series of hooks, pins, or posts have been used to contain collagen gels but such an addition might add unwanted complexity to the fabrication of the model [71,72]. Another strategy would be to chemically crosslink the ECM through various crosslinking methods available [46,73]. This could reduce the shrinkage but could potentially be harmful for the fibroblasts within if they are crosslinked to the ECM. A ruthenium/SPS crosslinking strategy was used to reinforce the mechanical stability of porcine heart ECM-derived hydrogels [74]. In that study human-induced pluripotent stem cell-derived cardiomyocytes were mixed into the ruthenium-reinforced porcine heart hydrogel and subsequently bioprinted using a UV-based printer. The cells remained viable for up to 14 days and were able to stretch and contract to Ca^+2^ exposure within the crosslinked hydrogel. One caveat is that with crosslinking the hydrogel, the stiffness also increases, which the cells sense and respond to accordingly [46]. The increased stiffness of the airways affects the epithelial cell differentiation, as seen with COPD primary epithelial cells grown on decellularized COPD bronchial scaffolds, which showed increased proliferation and retained a basal-cell phenotype when compared to cells grown on normal bronchial scaffolds [75,76].

In our model, fibroblasts persisted in most gels after 14 days of ALI culture, and research is needed to understand their role during this period and their influence on epithelial cell differentiation or ECM dynamics. Furthermore, in future studies, the ALI culture period could be extended to 28 days to create a fully differentiated human bronchial epithelium, containing basal, serous, Clara, goblet, and ciliated cells [66].

Air exposure of the apical layer has been shown not to negatively affect fibroblasts [77]. The ECM network analysis using TWOMBLI revealed little to no difference in matrix pattern metrics between the air-exposed and submerged culture models. However, one aspect of the ECM topology that was affected, either due to the contraction, prolonged culture, air exposure, or a combination, was the curvature—the parameter that describes the waviness of the ECM fibres. The curvature windows describe the wave pattens seen in the ECM fibres and together describe the wavelength and amplitude of the curves in the fibres [57,78]. All of the curvature windows showed an increase in the contracted hydrogels, both for porcine and human lung-derived ECM. The changes in fibre curvature give an indication of the overall structural changes in the ECM, i.e., waviness vs. straightness of the fibres and intra-fibre orientation. One possible explanation is that due to the compression forces exerted on the fibres by the cells, the fibres bent and buckled and generated a more wave-like pattern. These observed differences are likely due the variation in composition between different hydrogels, as demonstrated in a recent proteomic study highlighting differences in lung ECM composition between COPD and a control [39]. TWOMBLI, while relatively new, aids in understanding how crosslinking methods and cells modify ECM [46,78,79]. Continued use of this analysis approach will likely enhance our comprehension of ECM fibre characteristics and their impact.

Some limitations of our experimental approach must be recognized. Proteoglycans and growth factors are recognized to be lost or disrupted during the decellularization and ECM hydrogel preparation procedure [80,81], and the influence of these molecules on the rheological properties of the resultant hydrogel or epithelial cell differentiation are not currently known. The ECM hydrogels were made from the total lung, excluding the cartilaginous airways and major blood vessels but retaining the smallest airways and vessels, therefore they do contain BM proteins. This might mean that adding a basement membrane mimic might not be necessary or that another BM protein might be a better choice.

During the experimental timeframe, some hydrogels contracted significantly, becoming so small that sectioning them for measuring end points became problematic. Consequently, not all experimental conditions could be adequately represented in our subsequent analyses due to these challenges. While recognizing these limitations, there are many interesting applications and possible next steps to take with this model.

In COPD, immune cell infiltration within the small airways plays an important role, an aspect currently not present in our model [82]. Incorporating this element might be possible by integrating our model with perfusion models [83]. In order to fulfil this role effectively, the ECM hydrogel might require reinforcement to withstand mechanical stresses. Given that the lung undergoes 20,000 breathing cycles a day, incorporating this additional mechanical parameter into our model could be advantageous [84]. Combining a breathing perfusable system with our model would substantially advance our ability to model the airway in vitro [85,86]. This integration could bring us closer to replicating airway dynamics more accurately in an in vitro setting.

In conclusion, both the porcine and human lung ECM hydrogel primary cell co-culture models provide new opportunities for replicating the small-airway microenvironment and potentially other lung regions. With continuous refinement to ensure mechanical stability, these models now offer exciting opportunities for exploring human lung-derived cell responses across various ECM compositions by altering parameters such as stiffness or protein content. Expanding this model by including basolateral perfusion, immune cells, and mechanical stretching to mimic breathing would enhance its translational potential, progressively narrowing the divide between native lung tissues and in vitro model systems.

## Data Availability

Data are contained within the article and are available upon reasonable request.

## Supplementary Materials

The following supporting information can be downloaded at: https://www.mdpi.com/article/10.3390/bioengineering11090946/s1, Figure S1: Individual data points from the TWOMBLI matrix metrics analysis of 10 mg/mL porcine lung-derived hydrogel; Figure S2: Individual data points from the TWOMBLI matrix metrics analysis of 20 mg/mL porcine lung-derived hydrogel; Figure S3: Individual data points from the TWOMBLI matrix metrics analysis of control human lung-derived hydrogel; Figure S4: Individual data points from the TWOMBLI matrix metrics analysis of COPD human lung-derived hydrogel; Figure S5: Normality testing of 20 mg/mL porcine lung hydrogel matrix pattern metrics; Figure S6: Normality testing of control human lung hydrogel matrix pattern metrics; Figure S7: Normality testing of COPD human lung hydrogel matrix pattern metrics.

## References

[1] Quaderi S.A., Hurst J.R. (2018). The unmet global burden of COPD. Glob Health Epidemiol Genom.

[2] Eisner M.D., Anthonisen N., Coultas D., Kuenzli N., Perez-Padilla R., Postma D., Romieu I., Silverman E.K., Balmes J.R. (2010). An official American Thoracic Society public policy statement: Novel risk factors and the global burden of chronic obstructive pulmonary disease. Am. J. Respir. Crit. Care Med..

[3] (2016). Global, regional, and national incidence, prevalence, and years lived with disability for 310 diseases and injuries, 1990–2015: A systematic analysis for the Global Burden of Disease Study 2015. Lancet.

[4] Hogg J.C., Timens W. (2009). The pathology of chronic obstructive pulmonary disease. Annu. Rev. Pathol..

[5] Syamlal G., Doney B., Mazurek J.M. (2019). Chronic Obstructive Pulmonary Disease Prevalence Among Adults Who Have Never Smoked, by Industry and Occupation—United States, 2013–2017. MMWR Morb. Mortal Wkly. Rep..

[6] Agustí A., Celli B.R., Criner G.J., Halpin D., Anzueto A., Barnes P., Bourbeau J., Han M.K., Martinez F.J., Montes de Oca M. (2023). Global Initiative for Chronic Obstructive Lung Disease 2023 Report: GOLD Executive Summary. Eur. Respir. J..

[7] Hogg J.C., McDonough J.E., Gosselink J.V., Hayashi S. (2009). What drives the peripheral lung-remodeling process in chronic obstructive pulmonary disease?. Proc. Am. Thorac. Soc..

[8] Rabe K.F., Watz H. (2017). Chronic obstructive pulmonary disease. Lancet.

[9] Tam A., Wadsworth S., Dorscheid D., Man S.F., Sin D.D. (2011). The airway epithelium: More than just a structural barrier. Ther. Adv. Respir. Dis..

[10] Liu C., Li P., Zheng J., Wang Y., Wu W., Liu X. (2022). Role of necroptosis in airflow limitation in chronic obstructive pulmonary disease: Focus on small-airway disease and emphysema. Cell Death Discov..

[11] Pouwels S.D., Heijink I.H., Ten Hacken N.H., Vandenabeele P., Krysko D.V., Nawijn M.C., Van Oosterhout A.J. (2014). DAMPs activating innate and adaptive immune responses in COPD. Mucosal Immunol..

[12] Crystal R.G. (2014). Airway basal cells. The “smoking gun” of chronic obstructive pulmonary disease. Am. J. Respir. Crit. Care Med..

[13] Osei E.T., Brandsma C.-A., Noordhoek J.A., Timens W., Postma D., Heijink I. (2014). Crosstalk between epithelium and fibroblasts; implications for COPD. Eur. Respir. J..

[14] Fujita Y., Araya J., Ito S., Kobayashi K., Kosaka N., Yoshioka Y., Kadota T., Hara H., Kuwano K., Ochiya T. (2015). Suppression of autophagy by extracellular vesicles promotes myofibroblast differentiation in COPD pathogenesis. J. Extracell. Vesicles.

[15] Barnes P.J. (2019). Small airway fibrosis in COPD. Int. J. Biochem. Cell Biol..

[16] Hynes R.O. (2009). The extracellular matrix: Not just pretty fibrils. Science.

[17] Hynes R.O., Naba A. (2012). Overview of the Matrisome—An Inventory of Extracellular Matrix Constituents and Functions. Cold Spring Harb. Perspect. Biol..

[18] Jayadev R., Sherwood D.R. (2017). Basement membranes. Curr. Biol..

[19] Frantz C., Stewart K.M., Weaver V.M. (2010). The extracellular matrix at a glance. J. Cell Sci..

[20] Sun B. (2021). The mechanics of fibrillar collagen extracellular matrix. Cell Rep. Phys. Sci..

[21] Pomin V.H., Mulloy B. (2018). Glycosaminoglycans and proteoglycans. Pharmaceuticals.

[22] Burgess J.K., Mauad T., Tjin G., Karlsson J.C., Westergren-Thorsson G. (2016). The extracellular matrix—The under-recognized element in lung disease?. J. Pathol..

[23] Karsdal M.A., Nielsen M.J., Sand J.M., Henriksen K., Genovese F., Bay-Jensen A.C., Smith V., Adamkewicz J.I., Christiansen C., Leeming D.J. (2013). Extracellular matrix remodeling: The common denominator in connective tissue diseases. Possibilities for evaluation and current understanding of the matrix as more than a passive architecture, but a key player in tissue failure. Assay Drug Dev. Technol..

[24] Muncie J.M., Weaver V.M. (2018). The Physical and Biochemical Properties of the Extracellular Matrix Regulate Cell Fate. Curr. Top Dev. Biol..

[25] Wrench C., Baker J., Fenwick P., Donnelly L., Barnes P. (2018). Small airway fibroblasts from COPD patients are senescent and pro-fibrotic. Eur. Respir. J..

[26] Karakioulaki M., Papakonstantinou E., Stolz D. (2020). Extracellular matrix remodelling in COPD. Eur. Respir. Rev..

[27] Kim J.H., Schaible N., Hall J.K., Bartolák-Suki E., Deng Y., Herrmann J., Sonnenberg A., Behrsing H.P., Lutchen K.R., Krishnan R. (2023). Multiscale stiffness of human emphysematous precision cut lung slices. Sci. Adv..

[28] Parameswaran H., Majumdar A., Suki B. (2011). Linking microscopic spatial patterns of tissue destruction in emphysema to macroscopic decline in stiffness using a 3D computational model. PLoS Comput. Biol..

[29] Annoni R., Lanças T., Yukimatsu Tanigawa R., de Medeiros Matsushita M., de Morais Fernezlian S., Bruno A., Fernando Ferraz da Silva L., Roughley P.J., Battaglia S., Dolhnikoff M. (2012). Extracellular matrix composition in COPD. Eur. Respir. J..

[30] Kutluk H., Bastounis E.E., Constantinou I. (2023). Integration of Extracellular Matrices into Organ-on-Chip Systems. Adv. Healthc. Mater..

[31] Lutolf M.P., Raeber G.P., Zisch A.H., Tirelli N., Hubbell J.A. (2003). Cell-Responsive Synthetic Hydrogels. Adv. Mater..

[32] Hoffman A.S. (2012). Hydrogels for biomedical applications. Adv. Drug Deliv. Rev..

[33] Yuan T., Zhang L., Li K., Fan H., Fan Y., Liang J., Zhang X. (2014). Collagen hydrogel as an immunomodulatory scaffold in cartilage tissue engineering. J. Biomed. Mater. Res. Part B: Appl. Biomater..

[34] Rowley J.A., Madlambayan G., Mooney D.J. (1999). Alginate hydrogels as synthetic extracellular matrix materials. Biomaterials.

[35] Bhattarai N., Gunn J., Zhang M. (2010). Chitosan-based hydrogels for controlled, localized drug delivery. Adv. Drug Deliv. Rev..

[36] Reis L.A., Chiu L.L.Y., Liang Y., Hyunh K., Momen A., Radisic M. (2012). A peptide-modified chitosan–collagen hydrogel for cardiac cell culture and delivery. Acta Biomater..

[37] Sackett S., Tremmel D., Ma F., Feeney A.K., Maguire R.M., Brown M.E., Zhou Y., Li X., O’Brien C., Li L. (2018). Extracellular matrix scaffold and hydrogel derived from decellularized and delipidized human pancreas. Sci. Rep..

[38] Liguori G.R., Liguori T.T.A., de Moraes S.R., Sinkunas V., Terlizzi V., van Dongen J.A., Sharma P.K., Moreira L.F.P., Harmsen M.C. (2020). Molecular and Biomechanical Clues From Cardiac Tissue Decellularized Extracellular Matrix Drive Stromal Cell Plasticity. Front. Bioeng. Biotechnol..

[39] Hoffman E.T., Uhl F.E., Asarian L., Deng B., Becker C., Uriarte J.J., Downs I., Young B., Weiss D.J. (2023). Regional and disease specific human lung extracellular matrix composition. Biomaterials.

[40] de Hilster R.H.J., Sharma P.K., Jonker M.R., White E.S., Gercama E.A., Roobeek M., Timens W., Harmsen M.C., Hylkema M.N., Burgess J.K. (2020). Human lung extracellular matrix hydrogels resemble the stiffness and viscoelasticity of native lung tissue. Am. J. Physiol Lung Cell Mol. Physiol..

[41] Pouliot R.A., Link P.A., Mikhaiel N.S., Schneck M.B., Valentine M.S., Kamga Gninzeko F.J., Herbert J.A., Sakagami M., Heise R.L. (2016). Development and characterization of a naturally derived lung extracellular matrix hydrogel. J. Biomed. Mater. Res. A.

[42] Vieira Braga F.A., Kar G., Berg M., Carpaij O.A., Polanski K., Simon L.M., Brouwer S., Gomes T., Hesse L., Jiang J. (2019). A cellular census of human lungs identifies novel cell states in health and in asthma. Nat. Med..

[43] Heijink I.H., Kies P., Kauffman H.F., Postma D.S., van Oosterhout A.J., Vellenga E. (2007). Down-regulation of E-cadherin in human bronchial epithelial cells leads to epidermal growth factor receptor-dependent Th2 cell-promoting activity. J. Immunol..

[44] Dinesh Kumar N., Ter Ellen B.M., Bouma E.M., Troost B., van de Pol D.P., van der Ende-Metselaar H.H., van Gosliga D., Apperloo L., Carpaij O.A., van den Berge M. (2022). Moxidectin and ivermectin inhibit SARS-CoV-2 replication in Vero E6 cells but not in human primary bronchial epithelial cells. Antimicrob. Agents Chemother..

[45] Martinez-Garcia F.D., de Hilster R.H.J., Sharma P.K., Borghuis T., Hylkema M.N., Burgess J.K., Harmsen M.C. (2021). Architecture and Composition Dictate Viscoelastic Properties of Organ-Derived Extracellular Matrix Hydrogels. Polymers.

[46] Nizamoglu M., de Hilster R.H.J., Zhao F., Sharma P.K., Borghuis T., Harmsen M.C., Burgess J.K. (2022). An in vitro model of fibrosis using crosslinked native extracellular matrix-derived hydrogels to modulate biomechanics without changing composition. Acta Biomater.

[47] Sharma P., Busscher H., Terwee T., Koopmans S., van Kooten T. (2011). A comparative study on the viscoelastic properties of human and animal lenses. Exp. Eye Res..

[48] Noordhoek J.A., Postma D.S., Chong L.L., Menkema L., Kauffman H.F., Timens W., van Straaten J.F., van der Geld Y.M. (2005). Different modulation of decorin production by lung fibroblasts from patients with mild and severe emphysema. Copd.

[49] CORNING Certificate of Analysis—CORNING® COLLAGEN I, RAT TAIL. https://ecatalog.corning.com/life-sciences/b2c/US/en/Surfaces/Extracellular-Matrices-ECMs/Corning%C2%AE-Collagen/p/354236.

[50] Martinez-Garcia F.D., Valk M.M., Sharma P.K., Burgess J.K., Harmsen M.C. (2021). Adipose Tissue-Derived Stromal Cells Alter the Mechanical Stability and Viscoelastic Properties of Gelatine Methacryloyl Hydrogels. Int. J. Mol. Sci..

[51] Lai M., Lü B. (2012). Tissue preparation for microscopy and histology. Comprehensive Sampling and Sample Preparation.

[52] Tasena H., Timens W., van den Berge M., van Broekhuizen J., Kennedy B.K., Hylkema M.N., Brandsma C.A., Heijink I.H. (2022). MicroRNAs Associated with Chronic Mucus Hypersecretion in COPD Are Involved in Fibroblast-Epithelium Crosstalk. Cells.

[53] Spanjer A.I., Menzen M.H., Dijkstra A.E., van den Berge M., Boezen H.M., Nickle D.C., Sin D.D., Bossé Y., Brandsma C.-A., Timens W. (2016). A pro-inflammatory role for the Frizzled-8 receptor in chronic bronchitis. Thorax.

[54] Fischer A.H., Jacobson K.A., Rose J., Zeller R. (2008). Hematoxylin and eosin staining of tissue and cell sections. CSH Protoc..

[55] Schindelin J., Arganda-Carreras I., Frise E., Kaynig V., Longair M., Pietzsch T., Preibisch S., Rueden C., Saalfeld S., Schmid B. (2012). Fiji: An open-source platform for biological-image analysis. Nat. Methods.

[56] Ngassie M.L.K., De Vries M., Borghuis T., Timens W., Sin D.D., Nickle D., Joubert P., Horvatovich P., Marko-Varga G., Teske J.J. (2023). Age-associated differences in the human lung extracellular matrix. Am. J. Physiol.-Lung Cell. Mol. Physiol..

[57] Wershof E., Park D., Barry D.J., Jenkins R.P., Rullan A., Wilkins A., Schlegelmilch K., Roxanis I., Anderson K.I., Bates P.A. (2021). A FIJI macro for quantifying pattern in extracellular matrix. Life Sci. Alliance.

[58] Jiang D., Schaefer N., Chu H.W. (2018). Air-Liquid Interface Culture of Human and Mouse Airway Epithelial Cells. Methods Mol. Biol..

[59] Wu R., Sato G.H., Whitcutt M.J. (1986). Developing differentiated epithelial cell cultures: Airway epithelial cells. Fundam. Appl. Toxicol..

[60] Jain P., Rauer S.B., Möller M., Singh S. (2022). Mimicking the Natural Basement Membrane for Advanced Tissue Engineering. Biomacromolecules.

[61] Heijink I.H., de Bruin H.G., Dennebos R., Jonker M.R., Noordhoek J.A., Brandsma C.A., van den Berge M., Postma D.S. (2016). Cigarette smoke-induced epithelial expression of WNT-5B: Implications for COPD. Eur. Respir. J..

[62] Hohenester E., Yurchenco P.D. (2013). Laminins in basement membrane assembly. Cell Adh Migr..

[63] Dekkers B.G.J., Saad S.I., van Spelde L.J., Burgess J.K. (2021). Basement membranes in obstructive pulmonary diseases. Matrix Biol. Plus.

[64] Liesker J.J., Hacken N.H.T., Zeinstra-Smith M., Rutgers S.R., Postma D.S., Timens W. (2009). Reticular basement membrane in asthma and COPD: Similar thickness, yet different composition. Int. J. Chronic Obstr. Pulm. Dis..

[65] Kranenburg A.R., Willems-Widyastuti A., Mooi W.J., Sterk P.J., Alagappan V.K., de Boer W.I., Sharma H.S. (2006). Enhanced bronchial expression of extracellular matrix proteins in chronic obstructive pulmonary disease. Am. J. Clin. Pathol..

[66] Prytherch Z., Job C., Marshall H., Oreffo V., Foster M., BéruBé K. (2011). Tissue-Specific stem cell differentiation in an in vitro airway model. Macromol. Biosci..

[67] Handorf A.M., Zhou Y., Halanski M.A., Li W.J. (2015). Tissue stiffness dictates development, homeostasis, and disease progression. Organogenesis.

[68] Chaudhuri O., Gu L., Klumpers D., Darnell M., Bencherif S.A., Weaver J.C., Huebsch N., Lee H.P., Lippens E., Duda G.N. (2016). Hydrogels with tunable stress relaxation regulate stem cell fate and activity. Nat. Mater..

[69] Hinz B., McCulloch C.A., Coelho N.M. (2019). Mechanical regulation of myofibroblast phenoconversion and collagen contraction. Exp Cell Res.

[70] Bourke J.E., Li X., Foster S.R., Wee E., Dagher H., Ziogas J., Harris T., Bonacci J.V., Stewart A.G. (2011). Collagen remodelling by airway smooth muscle is resistant to steroids and β2-agonists. Eur. Respir. J..

[71] Hameed P., Manivasagam G. (2021). An overview of bio-actuation in collagen hydrogels: A mechanobiological phenomenon. Biophys. Rev..

[72] Zhang Q., Wang P., Fang X., Lin F., Fang J., Xiong C. (2022). Collagen gel contraction assays: From modelling wound healing to quantifying cellular interactions with three-dimensional extracellular matrices. Eur. J. Cell Biol..

[73] Sarrigiannidis S.O., Rey J.M., Dobre O., González-García C., Dalby M.J., Salmeron-Sanchez M. (2021). A tough act to follow: Collagen hydrogel modifications to improve mechanical and growth factor loading capabilities. Mater Today Bio..

[74] Kim H., Kang B., Cui X., Lee S.H., Lee K., Cho D.W., Hwang W., Woodfield T.B., Lim K.S., Jang J. (2021). Light-activated decellularized extracellular matrix-based bioinks for volumetric tissue analogs at the centimeter scale. Adv. Funct. Mater..

[75] Hedström U., Öberg L., Vaarala O., Dellgren G., Silverborn M., Bjermer L., Westergren-Thorsson G., Hallgren O., Zhou X. (2021). Impaired Differentiation of Chronic Obstructive Pulmonary Disease Bronchial Epithelial Cells Grown on Bronchial Scaffolds. Am. J. Respir. Cell Mol. Biol..

[76] Jones R.L., Noble P.B., Elliot J.G., James A.L. (2016). Airway remodelling in COPD: It’s not asthma!. Respirology.

[77] Toda S., Yokoi F., Yamada S., Yonemitsu N., Nishimura T., Watanabe K., Sugihara H. (2000). Air exposure promotes fibroblast growth with increased expression of mitogen-activated protein kinase cascade. Biochem. Biophys. Res. Commun..

[78] Nizamoglu M., Alleblas F., Koster T., Borghuis T., Vonk J.M., Thomas M.J., White E.S., Watson C.K., Timens W., Kasmi K.C.E. (2024). Three dimensional fibrotic extracellular matrix directs microenvironment fiber remodeling by fibroblasts. Acta Biomater..

[79] Wijsman P.C., Goorsenberg A.W., Keijzer N., d’Hooghe J.N., Ten Hacken N.H., Shah P.L., Weersink E.J., de Brito J.M., Costa N.d.S.X., Mauad T. (2024). Airway wall extracellular matrix changes induced by bronchial thermoplasty in severe asthma. J. Allergy Clin. Immunol..

[80] Crapo P.M., Gilbert T.W., Badylak S.F. (2011). An overview of tissue and whole organ decellularization processes. Biomaterials.

[81] Mendoza-Novelo B., Avila E.E., Cauich-Rodríguez J.V., Jorge-Herrero E., Rojo F.J., Guinea G.V., Mata-Mata J.L. (2011). Decellularization of pericardial tissue and its impact on tensile viscoelasticity and glycosaminoglycan content. Acta Biomater..

[82] Higham A., Quinn A.M., Cançado J.E.D., Singh D. (2019). The pathology of small airways disease in COPD: Historical aspects and future directions. Respir. Res..

[83] Baker B.M., Trappmann B., Stapleton S.C., Toro E., Chen C.S. (2013). Microfluidics embedded within extracellular matrix to define vascular architectures and pattern diffusive gradients. Lab A Chip.

[84] Vindin H., Mithieux S.M., Weiss A.S. (2019). Elastin architecture. Matrix Biol..

[85] Huh D.D. (2015). A human breathing lung-on-a-chip. Ann. Am. Thorac. Soc..

[86] Zamprogno P., Wüthrich S., Achenbach S., Thoma G., Stucki J.D., Hobi N., Schneider-Daum N., Lehr C.M., Huwer H., Geiser T. (2021). Second-generation lung-on-a-chip with an array of stretchable alveoli made with a biological membrane. Commun. Biol..

