# Supplementary material for: A 3D Epithelial–Mesenchymal Co-Culture Model of the Airway Wall Using Native Lung Extracellular Matrix
Source: Bioengineering (Basel). 2024 Sep 21;11(9):946. doi: 10.3390/bioengineering11090946 (PMC11428669; doi:10.3390/bioengineering11090946)
Supplement: Supplementary file 1 [file bioengineering-11-00946-s001.zip › bioengineering-3165969-supplementary.pdf]

bioengineering

MDPI

Supplementary Materials

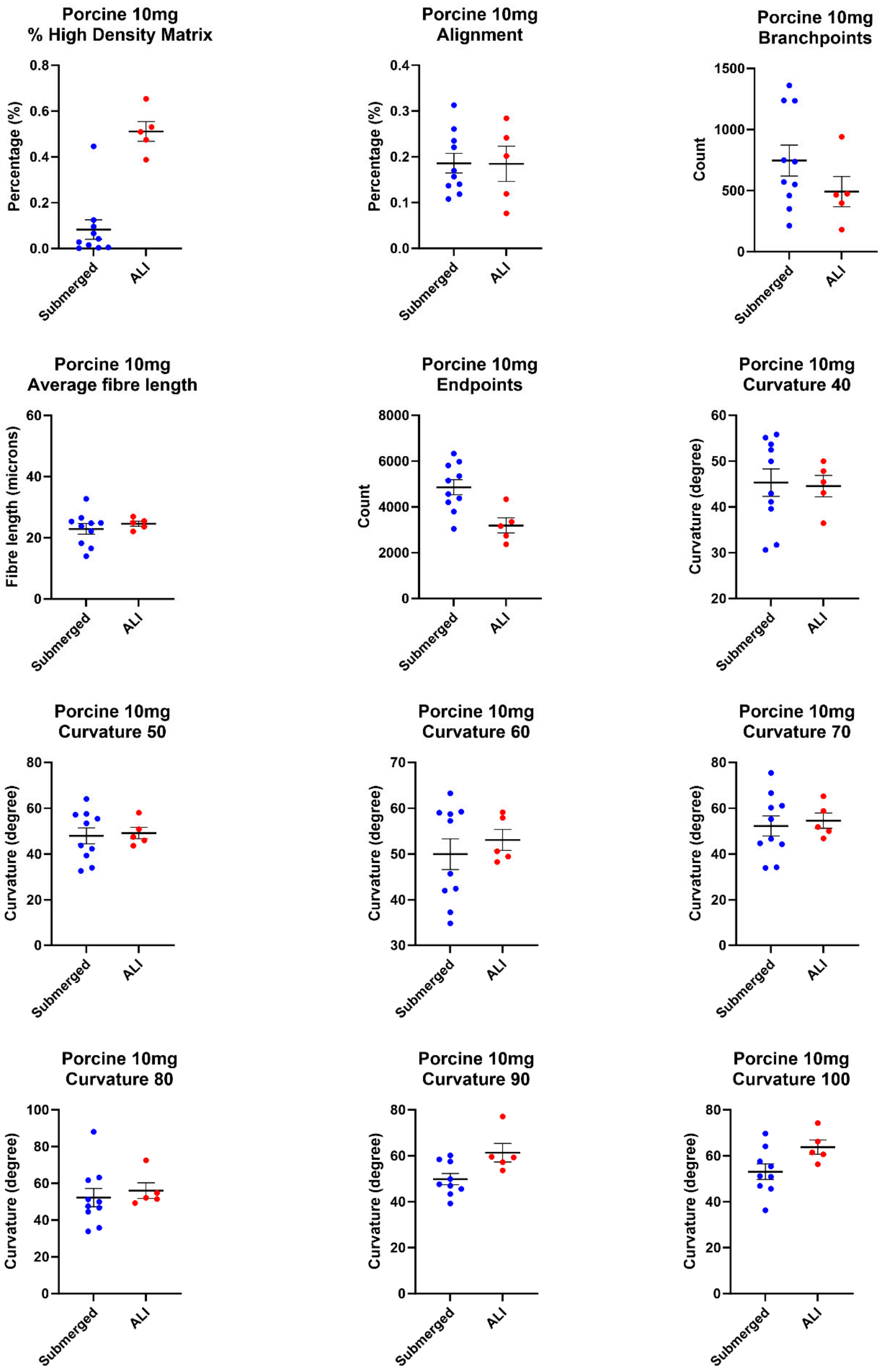

Figure S1. Individual data points from the TWOMBLI matrix metrics analysis of 10 mg/mL porcine lung derived hydrogel. Graphs generated from the results of the TWOMBLI analysis for the matrix metrics: percentage high density matrix (HDM), fibre alignment, average fibre length, fibre branchpoints, endpoints and all the curvature windows. Data is presented as individual values, mean and standard error of the mean. ALI: air liquid interface.

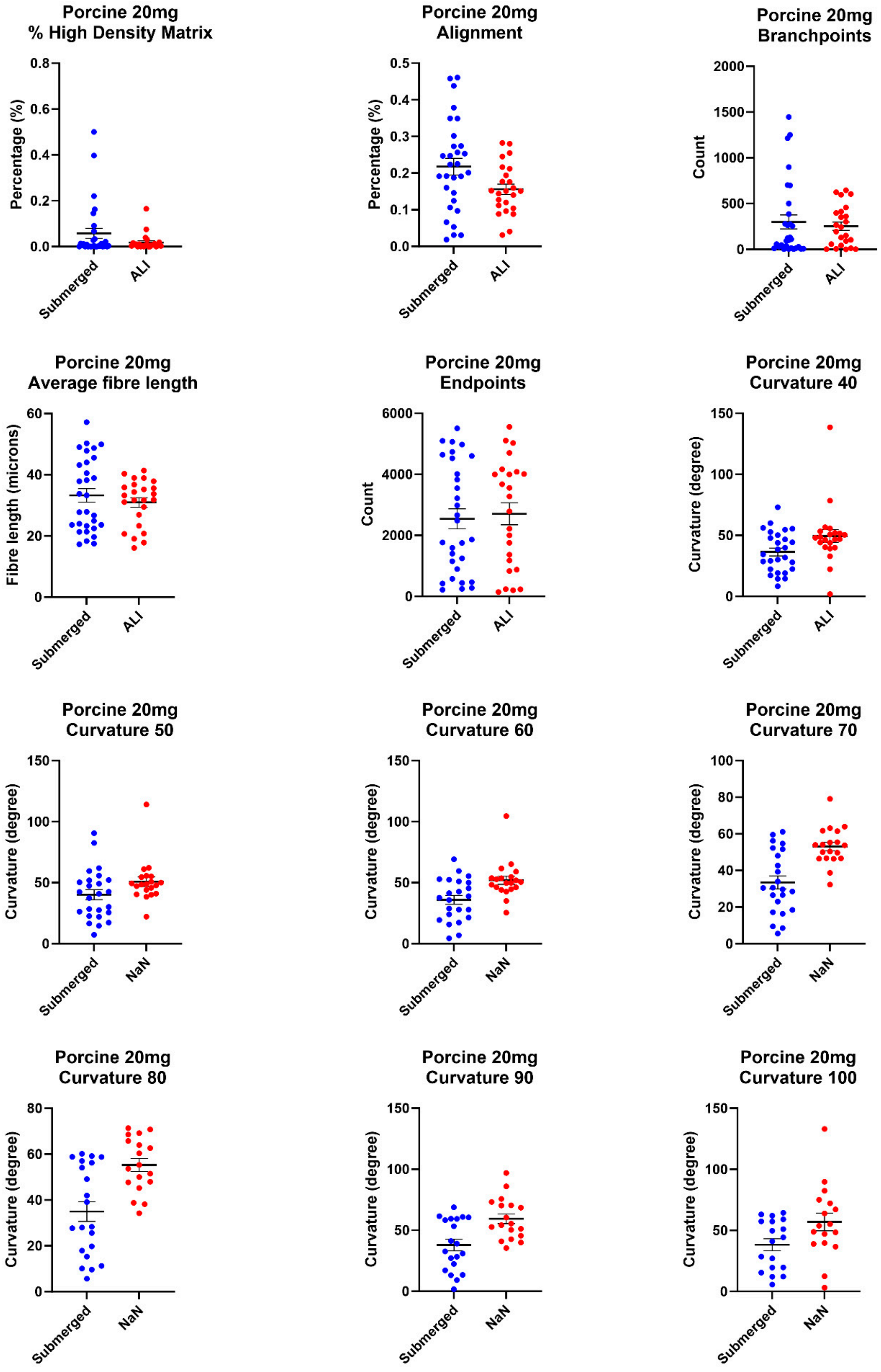

Figure S2. Individual data points from the TWOMBLI matrix metrics analysis of 20 mg/mL porcine lung derived hydrogel. Graphs generated from the results of the TWOMBLI analysis for the matrix metrics: percentage high density matrix (HDM), fibre alignment, average fibre length, fibre branchpoints, endpoints and all the curvature windows. Data is presented as individual values, mean and standard error of the mean. ALI: air liquid interface.

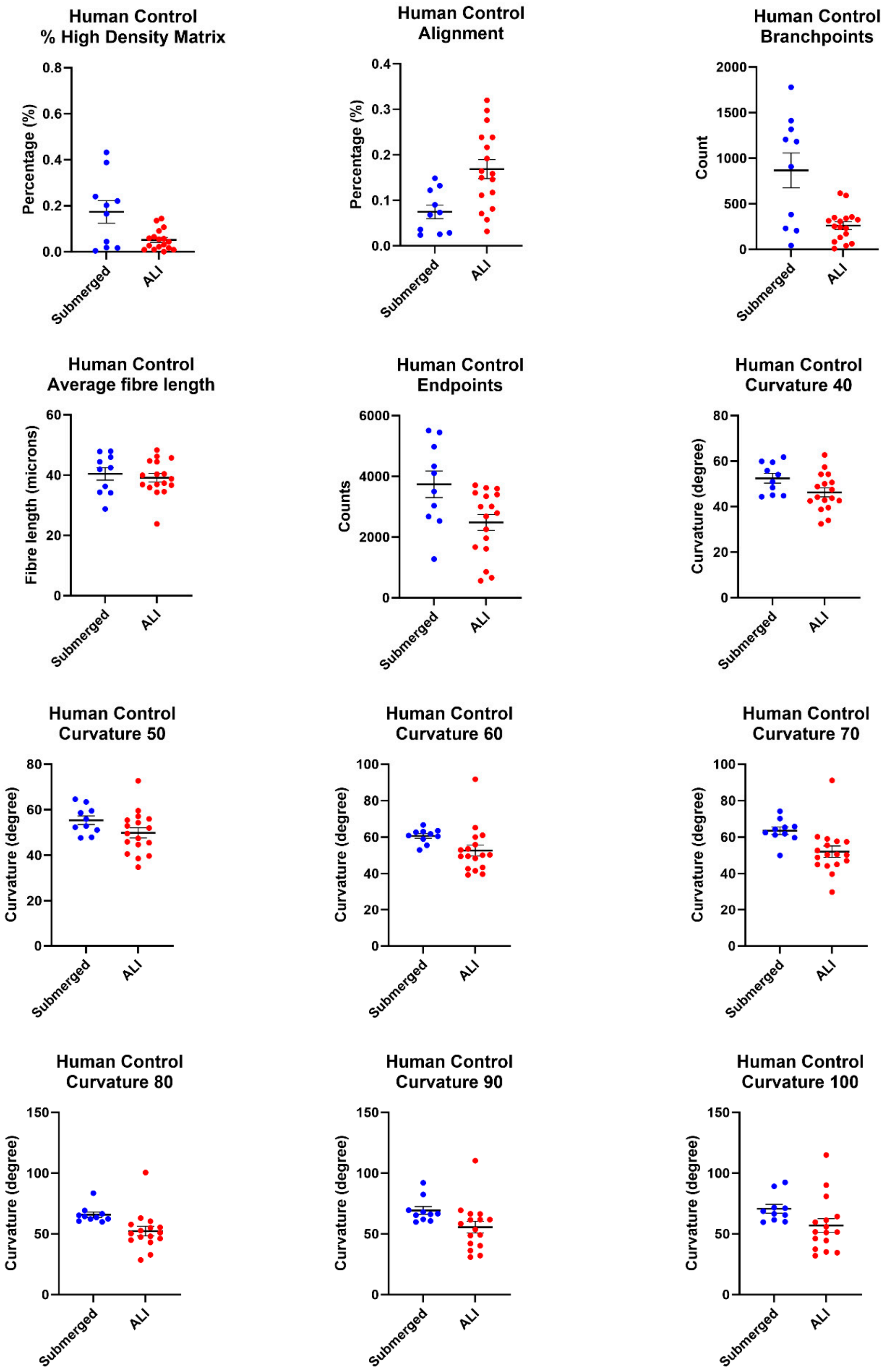

Figure S3. Individual data points from the TWOMBLI matrix metrics analysis of control human lung derived hydrogel. Graphs generated from the results of the TWOMBLI analysis for the matrix metrics: percentage high density matrix (HDM), fibre alignment, average fibre length, fibre branchpoints, endpoints and all the curvature windows. Data is presented as individual values, mean and standard error of the mean. ALI: air liquid interface.

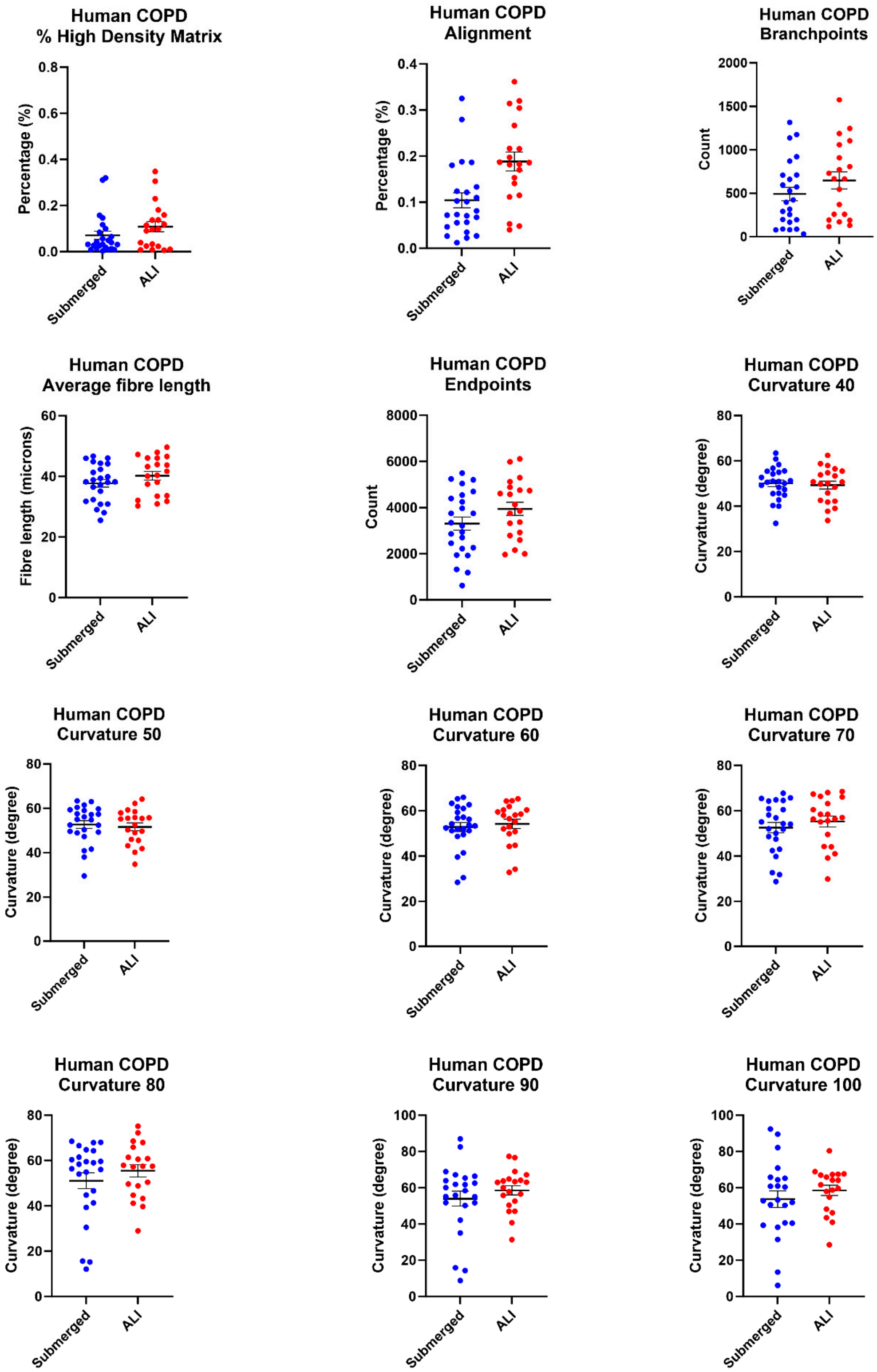

Figure S4. Individual data points from the TWOMBLI matrix metrics analysis of COPD human lung derived hydrogel. Graphs generated from the results of the TWOMBLI analysis for the matrix metrics: percentage high density matrix (HDM), fibre alignment average fibre length, fibre branchpoints, endpoints and all the curvature windows. Data is presented as individual values, mean and standard error of the mean. ALI: air liquid interface.

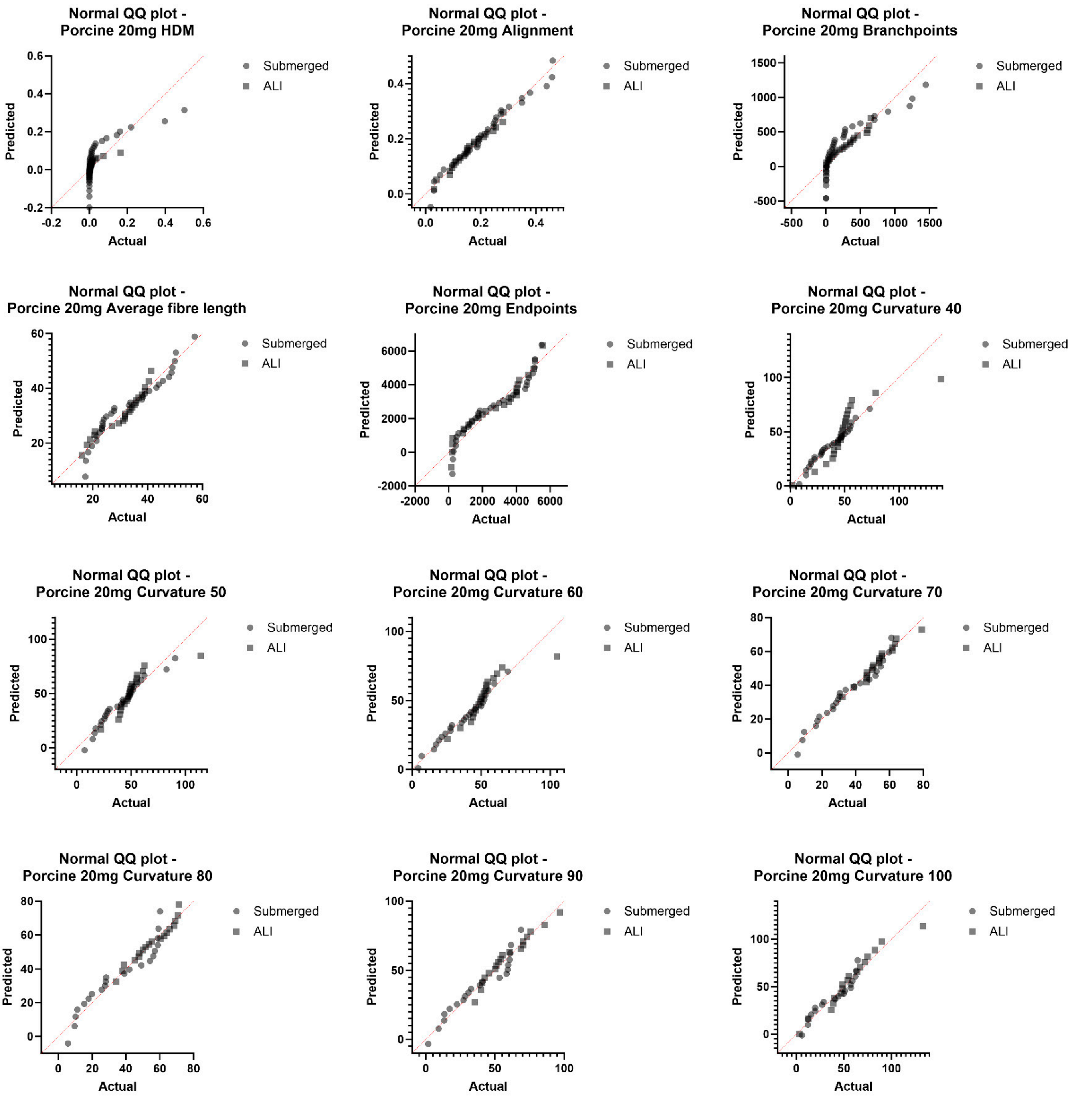

Figure S5. Normality testing 20mg/mL porcine lung hydrogel matrix pattern metrics. Q-Q plots for the normality assessments of the data analysed using mixed-model analysis. This includes all matrix pattern metrics: percentage high density matrix (HDM), fibre alignment average fibre length, fibre branchpoints, endpoints and all the curvature windows (Curvature 40, 50, 60, 70, 80, 90 and 100). ALI: air liquid interface.

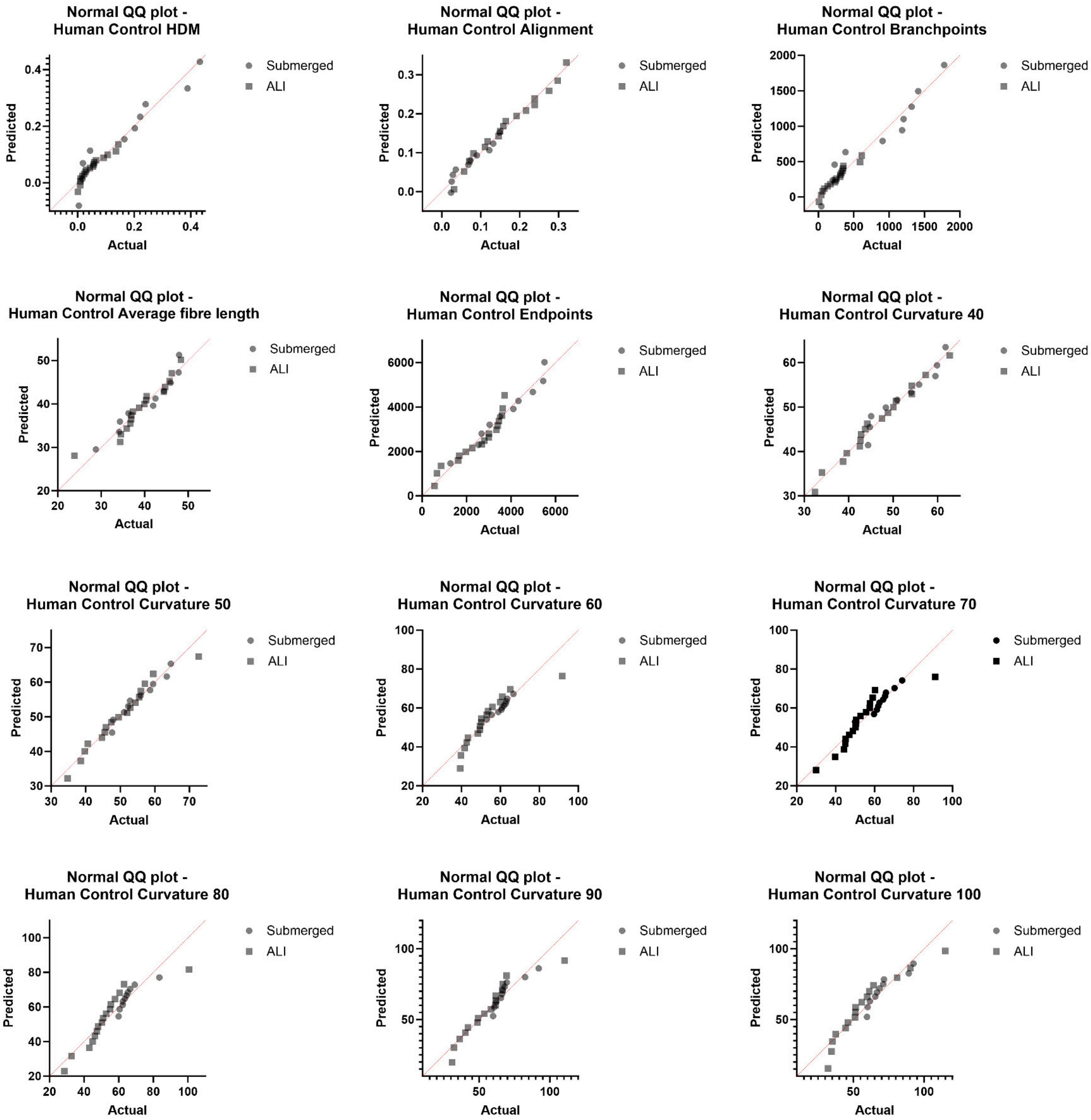

Figure S6. Normality testing control human lung hydrogel matrix pattern metrics. Q-Q plots for the normality assessments of the data analysed using mixed-model analysis. This includes all matrix pattern metrics: percentage high density matrix (HDM), fibre alignment, average fibre length, fibre branchpoints, endpoints and all the curvature windows (Curvature 40, 50, 60, 70, 80, 90 and 100). ALI: air liquid interface.

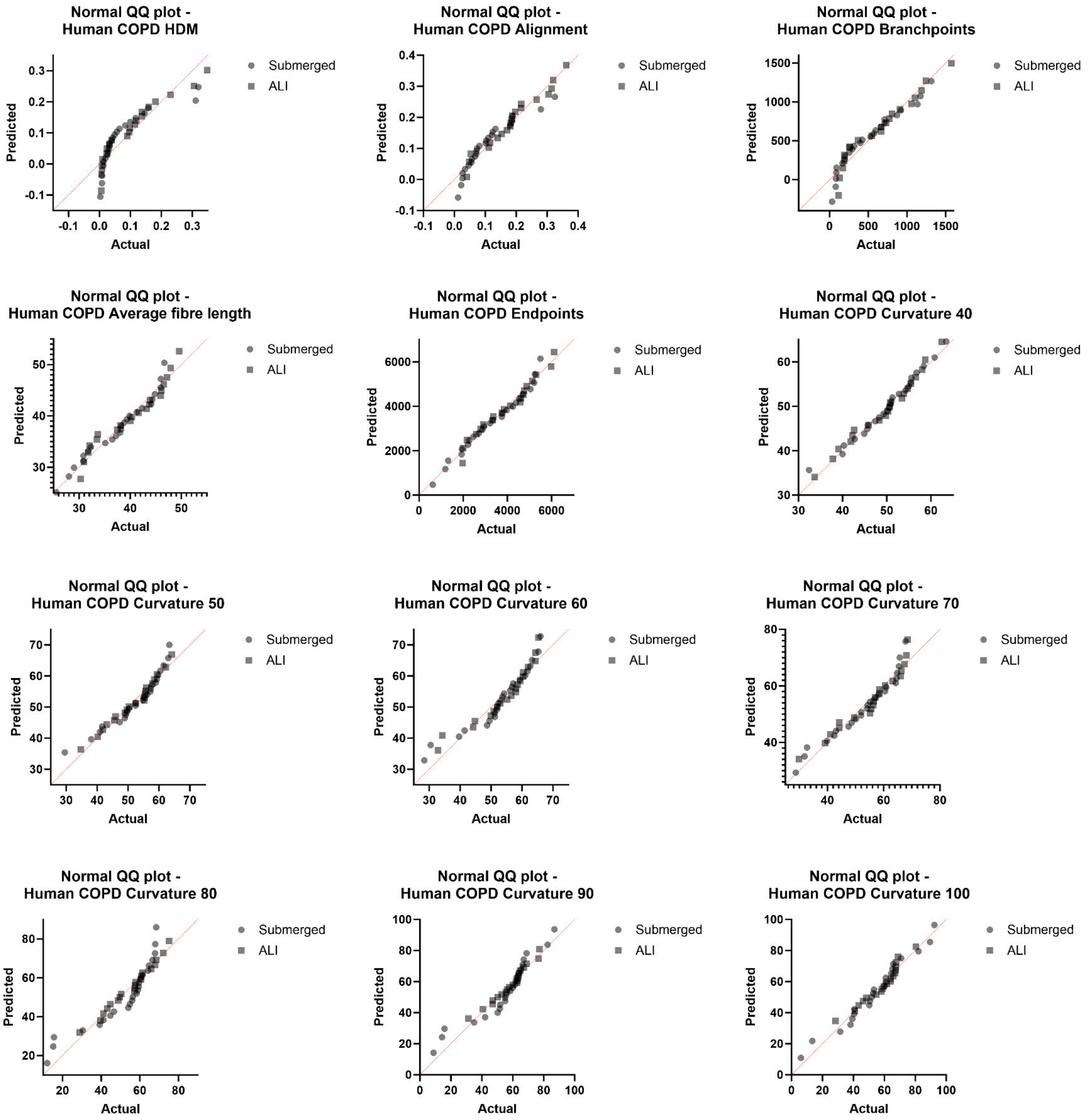

Figure S7. Normality testing COPD human lung hydrogel matrix pattern metrics. Q-Q plots for the normality assessments of the data analysed using mixed-model analysis. This includes all matrix pattern metrics: percentage high density matrix (HDM), fibre alignment, average fibre length, fibre branchpoints, endpoints and all the curvature windows (Curvature 40, 50, 60, 70, 80, 90 and 100). ALI: air liquid interface.
